# Combining laser microdissection and RNA-seq to chart the transcriptional landscape of fungal development

**DOI:** 10.1186/1471-2164-13-511

**Published:** 2012-09-27

**Authors:** Ines Teichert, Gabriele Wolff, Ulrich Kück, Minou Nowrousian

**Affiliations:** 1 Lehrstuhl für Allgemeine und Molekulare Botanik, Ruhr-Universität Bochum, Bochum, 44780, Germany

## Abstract

**Background:**

During sexual development, filamentous ascomycetes form complex, three-dimensional fruiting bodies for the protection and dispersal of sexual spores. Fruiting bodies contain a number of cell types not found in vegetative mycelium, and these morphological differences are thought to be mediated by changes in gene expression. However, little is known about the spatial distribution of gene expression in fungal development. Here, we used laser microdissection (LM) and RNA-seq to determine gene expression patterns in young fruiting bodies (protoperithecia) and non-reproductive mycelia of the ascomycete *Sordaria macrospora*.

**Results:**

Quantitative analysis showed major differences in the gene expression patterns between protoperithecia and total mycelium. Among the genes strongly up-regulated in protoperithecia were the pheromone precursor genes *ppg1* and *ppg2*. The up-regulation was confirmed by fluorescence microscopy of *egfp* expression under the control of *ppg1* regulatory sequences. RNA-seq analysis of protoperithecia from the sterile mutant pro1 showed that many genes that are differentially regulated in these structures are under the genetic control of transcription factor PRO1.

**Conclusions:**

We have generated transcriptional profiles of young fungal sexual structures using a combination of LM and RNA-seq. This allowed a high spatial resolution and sensitivity, and yielded a detailed picture of gene expression during development. Our data revealed significant differences in gene expression between protoperithecia and non-reproductive mycelia, and showed that the transcription factor PRO1 is involved in the regulation of many genes expressed specifically in sexual structures. The LM/RNA-seq approach will also be relevant to other eukaryotic systems in which multicellular development is investigated.

## Background

Fungi are a large group of eukaryotes consisting of a great number of species with a worldwide distribution and great impact on ecology and human society [1,2]. Fungi comprise both unicellular and multicellular species (yeasts and filamentous fungi, respectively), as well as species capable of both growth forms (dimorphic fungi). All filamentous fungi form a network of vegetative hyphae, called mycelium, that usually grows within or on substrates to acquire nutrients. In addition, many filamentous fungi are capable of developing complex, three-dimensional structures for the generation, protection, and dispersal of spores. Examples are conidiophores for the production of vegetative spores, and fruiting bodies for the production of sexual spores. Fruiting bodies are produced by many ascomycetes and basidiomycetes, and contain a number of specialized cell types that are not present in the vegetative mycelium [3,4]. The differentiation of these cell types is thought to be orchestrated by spatio-temporal changes in gene expression under the control of regulatory genetic networks. To address the question of developmental regulation of gene expression on a larger scale, several expression studies have been performed with the ascomycetes *Gibberella zeae* (anamorph *Fusarium graminearum*), *Neurospora crassa*, and *Sordaria macrospora*, all of which belong to the Sordariomycetes and form flask-like fruiting bodies called perithecia. Expression analyses were carried out using high-throughput methods, such as EST sequencing and microarray hybridization [5-13]. In most of the analyses, either time courses of developing mycelia and fruiting bodies were analyzed, or wild-type strains were compared to developmental mutants at certain time points during development. However, the tissues used in these studies usually contained cells from fruiting bodies and vegetative mycelium in varying proportions. One reason for this is that fruiting bodies in ascomycetes are often surrounded by or embedded in vegetative mycelium, from which they are difficult to separate; another reason is that especially the early stages of fruiting body development are quite small (< 50 μm), and even if collected would yield low amounts of material for RNA preparation and subsequent detection methods, such as microarray hybridizations. Therefore, little information is available regarding the spatial control of gene expression patterns, especially for the early stages of fruiting body development.

Laser microdissection (LM) can be used to isolate specific structures consisting of a few cells from samples mounted on microscope slides. LM has been used to isolate cells from animal and plant tissues, and in the case of fungi to study the growth of phytopathogenic or symbiotic species *in planta* and for the analysis of gene expression differences in single, neighboring hyphae [14-23]. Here, we have established an LM protocol for isolating fruiting body precursors called protoperithecia (young fruiting bodies that are more-or-less spherical without a differentiated neck) from the filamentous fungus *S. macrospora*. This ascomycete is a model system for the analysis of fungal sexual development and cell differentiation [24,25]. The genome was sequenced recently using next-generation sequencing techniques [26], and a number of developmental mutants have already been characterized by classical complementation analyses or by the sequencing of mutant genomes [27,28]. Prior to the availability of the *S. macrospora* genome sequence, we had already conducted large-scale expression analyses using cross-species microarray hybridizations with microarrays based on *N. crassa* cDNAs or oligonucleotides to study gene expression during development in the wild-type and several sterile mutants [8-11]. However, these analyses were limited in sensitivity because less conserved genes give low signal-to-noise ratios in the cross-species array hybridizations. With the genome sequence available, RNA-seq is now the method of choice for large-scale expression analysis. The unprecedented sequencing depths that can be achieved using next-generation sequencing techniques to sequence cDNAs allows much higher sensitivity than microarray hybridization, and the RNA-seq data can also be used for annotation purposes [29-31].

RNA-seq has been used in combination with LM to study gene expression in apical meristems and female gametophytes of *Arabidopsis thaliana*, in ripening tomato fruits, and in nucleus accumbens neurons in rats [32-35]; however, the combination of LM and RNA-seq has not yet been applied to the analysis of fungal organ-specific transcriptomes. Therefore, in the present study we established an LM protocol for isolating protoperithecia of *S. macrospora*, and used amplified RNA from the microdissected samples in subsequent RNA-seq analysis. Based on the RNA-seq data, we modeled untranslated regions (UTRs) for more than 50% of the predicted *S. macrospora* genes, and improved the annotation of roughly 1000 genes. We then compared gene expression patterns in wild-type protoperithecia to those of non-reproductive mycelium from the wild-type, as well as to protoperithecia from the developmental mutant pro1. The sterility of the pro1 mutant is caused by deletion of the transcription factor gene *pro1*[36]; and one aim of the study was to identify genes that are differentially regulated in protoperithecia, depending on or independent of the PRO1 transcription factor.

## Results

### Laser microdissection of protoperithecia and RNA-seq analysis

**Figure 1:**
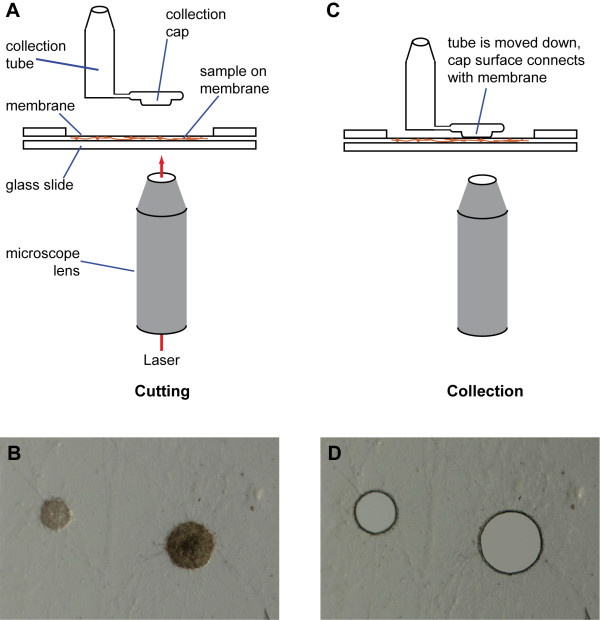
**Laser microdissection of protoperithecia.** Mycelia were grown on special membrane slides and fixed in ethanol. After drying of the slides, samples were covered with a glass slide (**A**) and visualized on an inverted microscope (**B**). Selected regions containing protoperithecia were cut with a UV laser through the microscope lens. To collect the cut out regions, the cap of a special collection tube was lowered onto the sample (**C**) where the membrane (with the sample attached) stuck to the cap and could be lifted off when the cap was raised again. Effective collection was indicated by corresponding holes in the samples (**D**).

For LM, strains were grown directly on slides for fixation and dissection *in situ*. To allow protoperithecial development, slides had to be covered with a thin layer of agar that did not interfere with the laser sectioning. Samples were fixed in ethanol, and microdissection was performed with a CellCut Plus system (see Methods and Figure 1). Approximately 100–300 protoperithecia with a diameter of ~20 μm were collected from each slide, pooled in a collection tube, and RNA was extracted from the collected protoperithecia with the PicoPure kit. It was then tested whether transcripts of protein-coding genes could be detected in the protoperithecial RNA samples by quantitative real time PCR (qRT-PCR). Expression was detectable for several genes that were analyzed in microdissected samples from the wild-type; but the amount of RNA was not sufficient for RNA-seq analysis. Therefore, two rounds of linear RNA amplification were performed based on cDNA generation and *in vitro* transcription [37,38] to obtain polyA-tailed RNA in the amounts required for Illumina/Solexa library generation. This linear amplification method has been shown previously to preserve relative transcript amounts within samples and is used in many applications including target generation for microarray hybridization [39]. The amplified RNAs from microdissected protoperithecia from the wild-type as well as from mutant pro1 were used for RNA-seq analysis. The pro1 mutant lacks the gene for the transcription factor PRO1, which is essential for sexual development; thus, the mutant is able to form protoperithecia but not mature fruiting bodies [36,40]. Therefore, genes that are differentially regulated in pro1 protoperithecia compared to those of the wild-type are direct or indirect targets of PRO1, and some of these genes might be required for fruiting body formation. 

In addition to the RNAs from microdissected samples, we used RNAs from total sexual or total vegetative mycelium. Total sexual mycelium (hereafter called “sexual mycelium”) was grown as a surface culture in liquid medium, which is the standard condition for fruiting body formation and RNA extraction from *S. macrospora*. RNA was extracted from the complete samples comprising protoperithecia as well as the surrounding vegetative hyphae [10]; with the latter making up the bulk of the sample. Samples for total vegetative mycelium (hereafter called “vegetative mycelium”) were grown submerged in liquid cultures to prevent the formation of any sexual structures [41]. In contrast to previous microarray analyses, we used pooled RNA from samples grown in cornmeal medium and defined medium (Table 1). Both media allow fruiting body formation in surface cultures and only vegetative growth in shaken cultures. We used pooled RNAs for two reasons: First, pooling allowed us to focus on genes that are differentially expressed during fruiting body formation independent of the growth medium, because differential gene expression that occurred only in one medium would be “quenched” in a pooled sample. Second, the use of different growth conditions should give a higher number of expressed genes, which was important because we wanted to use the data not only for expression analysis, but also for annotation purposes. Two biologically independent replications of each condition were used for RNA-seq by Illumina/Solexa sequencing, and 9–76 million single reads were obtained for each replicate (Table 1). Reads were cleaned using custom-made Perl scripts and mapped to the reference genome with the splice mapper Tophat [42], resulting in 9–66 million mapped reads per sample. The percentage of reads that mapped to the reference sequence was lower for the microdissected samples than for the samples derived from vegetative or sexual mycelium (Table 1); however, the overall number of reads obtained for the samples was still high, and this deep sequencing approach allowed a subsequent analysis of gene expression across the complete genome. 

**Table 1:** Summary of sequence reads generated in this study

**condition**	**sample**	**read length in bases**	**no. of reads**	**no. of reads mappedto reference genome**	**% of reads mappedto reference genome**
vegetative mycelium^1^	SM1	40	19,709,656	18,881,235	95.7
	SM6	101	76,664,943	66,365,082	86.5
sexual mycelium^2^	SM2	35	9,445,238	9,003,807	95.3
	SM7	101	68,217,654	60,021,065	87.9
wild-type protoperithecia^3^	SM4	101	31,837,927	24,722,821	77.6
	SM5	101	34,225,766	27,430,438	80.1
pro1 protoperithecia^3^	SM8	100	52,257,489	30,321,586	58.0
	SM9	100	53,996,808	25,265,576	46.8

The total number of reads is the number of reads after cleaning. Each sample represents an independent biological replicate.

^1^Equal amounts of RNA from the following growth conditions (independent biological replicates for each growth condition) were pooled: growth on cornmeal medium for 3d, 4d and 5d, growth on defined medium for 3d, 4d, and 5d; all in shaken cultures.

^2^Equal amounts of RNA from the following growth conditions (independent biological replicates for each growth condition) were pooled: growth on cornmeal medium for 3d, 4d and 5d, growth on defined medium for 3d, 4d, and 5d; all in surface cultures.

^3^Laser microdissected samples.

### Improving the *S. macrospora* genome annotation based on RNA-seq data

Mapped RNA-seq reads were visualized in the genome browser Artemis [43]. Though the reads from vegetative and sexual mycelium were evenly distributed along the transcripts, the reads derived from microdissected samples were clustered towards the 3’ end of most transcripts, an example is shown for *pro41* in Additional file 1 Figure S1. This 3’ bias was expected, because each round of RNA amplification leads to some loss at the 5’ end [39]. In addition, any UV damage during microdissection that might lead to strand cleavage would cause loss of the 5’ portion of the corresponding RNA because reverse transcription was based on polyA tails. In general, a 3’ bias does not hinder quantitative analysis as long as the relative amount of RNA in each sample is preserved, as it does not matter for general quantitation whether the reads from a transcript are evenly spread out or clustered towards one end. However, due to the 3’ bias, many of the reads from the protoperithecial LM samples mapped to the 3’ untranslated regions (UTRs), and therefore would only be taken into account when the 3’ UTR is annotated in the genome sequence. When we started the study, UTRs were annotated for only two *S. macrospora* genes. Therefore, we used the RNA-seq data to model UTRs and improve the exon-intron structures of the predicted *S. macrospora* genes in order to make full use of the RNA-seq data in subsequent quantitative expression analyses.

Based on the RNA-seq data, we were able to determine both UTRs for 48% of all predicted genes, and at least one UTR (5’ or 3’) for another 25% of all genes (Table 2, Additional file 1 Method S1 and Additional file 1 Figure S2). On average, 3’ UTRs are somewhat longer than 5’ UTRs with a median of 155 and 259 bases, respectively ( Additional file 1 Figure S1). These results are similar to those from an RNA-seq study of *Aspergillus oryzae*, in which median UTR lengths of 107 and 156 bases were found for the 5’ and 3’ UTR, respectively [44]. In addition to UTR modeling, ~1,000 *S. macrospora* gene models were improved or newly annotated based on spliced transcripts in the RNA-seq reads ( Additional file 1 Method S2 and Additional file 1 Figure S3), and these data are publicly available in genome version 2, (acc. no. CABT02000001-CABT02001583). Intron predictions based on RNA-seq reads recovered known introns that were verified experimentally in previous analyses, confirming that the RNA-seq-based gene model predictions are reliable. 

**Table 2:** UTR predictions for annotated genes

	**no. of genes**	**% of genes**
both UTRs found	4842	48.0
only 5’ UTR found	813	8.1
only 3’ UTR found	1707	16.9
no UTR found	2731	27.1

### Overview of gene expression across the *S. macrospora* genome

To obtain an overview of the genome-wide expression as represented by the RNA-seq data, reads that mapped to exons of predicted mRNAs as well as reads that mapped to intergenic regions or introns were counted based on the improved annotation using custom-made Perl scripts ( Additional file 1 Figure S4). The majority of reads mapped to annotated exons of protein-coding genes ( Additional file 1 Figure S5). This percentage was even higher for the protoperithecial samples, most likely because the two rounds of RNA amplification were based on polyA-dependent reverse transcription constituting an even stronger selection for protein-coding mRNAs. The true percentage of reads that map to intergenic regions might be even lower, because some genes are most likely still missing in the current annotation and not all UTRs for all predicted genes have been annotated yet (see previous section). Therefore, reads mapping to those regions would erroneously be counted as mapping to intergenic regions. A small percentage of reads mapped to introns and might represent incompletely processed transcripts or alternative splicing events, although an analysis of splice sites that were predicted by Tophat showed that, for the majority of genes, no significant alternative splicing could be detected in the different samples (data not shown).

Overall, only 764 predicted genes had no reads map to them in at least one condition ( Additional file 1 Figure S6); thus, the majority of genes were expressed in at least one of the four conditions investigated (sexual mycelium, vegetative mycelium, wild-type protoperithecia and pro1 protoperithecia). The majority (295 genes) of the 764 genes were not expressed in wild-type protoperithecia, but were expressed in the other three conditions including protoperithecia from mutant pro1. This finding might indicate that pro1 protoperithecia retain, at least to some degree, properties of non-reproductive mycelia, including the expression of genes that are not required in wild-type protoperithecia. In summary, expression was detected for more than 90% of all annotated genes (version 02) in at least one condition, confirming the high sensitivity of this deep-sequencing approach.

### Gene expression in protoperithecia and non-reproductive mycelia

For a quantitative analysis of gene expression in the different samples (vegetative and sexual mycelium, and protoperithecia from wild-type and pro1), sequence reads that mapped to predicted genes were counted using custom-made Perl scripts ( Additional file 1 Figure S4) and used for quantitative analysis. Results from LOX [45] and “classical analysis” [10] agreed best with the results from other methods, therefore these approaches were chosen for the final analysis (see Methods and Additional file 2). qRT-PCR was used to determine the expression of 17 genes in microdissected samples of wild-type protoperithecia without RNA amplification, and the results were compared to the RNA-seq data. In addition, qRT-PCR results and RNA-seq results were compared for gene expression in vegetative mycelium versus sexual mycelium ( Additional file 1 Figure S7). The overall results agree well, and tendencies (up- or down-regulation) are conserved with both methods.

**Figure 2:**
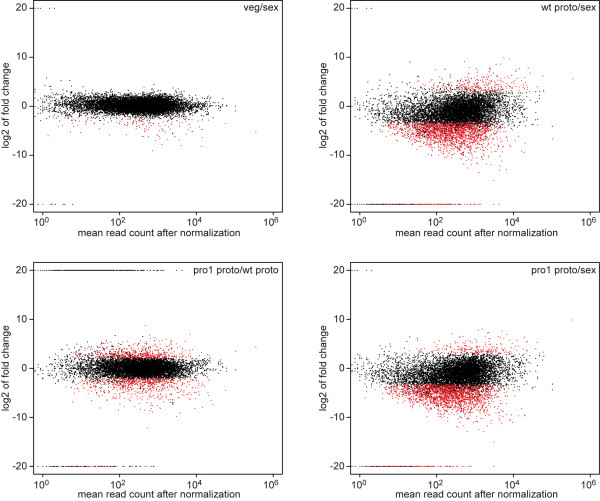
**MA-plots for gene expressiondata from different comparisons.** Log_2_ of fold ratios (M) were plotted against the average read counts (A) for the respective locus tag. The ratios were from the LOX analysis, the plots from the classic analysis look similar (data not shown). The log_2_ of ratios in which the denominator was zero were set to 20, and the log_2_ of ratios in which the numerator was zero were set to −20.

MA-plots of gene expression comparing the different samples showed that sexual mycelium is much more similar to vegetative mycelium than to protoperithecia from the wild-type or mutant pro1, and that the mutant and wild-type protoperithecia differ strongly from each other with respect to gene expression (Figure 2, Table 3, Additional file 2). The largest numbers of differentially regulated genes were those that are downregulated in wild-type or pro1 protoperithecia compared to sexual mycelium; however, some of these genes might be false-positives due to not all 3’ UTRs being annotated yet. In those cases, genes in protoperithecia samples might appear to be not expressed because most of the reads map to the 3’ ends of the genes and would not be counted for a gene if the 3’ UTR is not annotated correctly. This hypothesis is supported by the fact that the percentage of genes with annotated 3’ UTRs is lower among the genes that appear to be down-regulated in protoperithecia (Table 3). However, this problem does not occur in the comparison of the two protoperithecial samples from the wild-type and mutant pro1, because any bias would concern both samples equally. Therefore, the high number of differentially expressed genes in the comparison of wild-type and pro1 protoperithecia with almost equal numbers of up- and down-regulated genes most likely represents true differences in gene expression. The same is true for genes that are upregulated in protoperithecia compared to sexual mycelium, because this can not be overestimated by missing 3’ UTRs. Therefore, even when not taking into account the high number of putatively down-regulated genes in protoperithecia versus total mycelium, the data indicate that the differences between wild-type and pro1 protoperithecia as well as between protoperithecia and total mycelium (sexual and vegetative) are much more pronounced than between the different mycelial samples. This finding is consistent with the hypothesis that the morphological changes that occur during fruiting body formation are mediated by drastic changes in gene expression at the level of transcription.

**Table 3:** Comparison of gene expression in different samples

**comparison**	**upregulated**	**with 3’ UTR**^**1**^	**downregulated**	**with 3’ UTR**^**1**^
veg / sex	6	83%	117	72%
wt proto / sex	284	88%	2305	39%
pro1 proto / sex	222	85%	2292	39%
pro1 proto / wt proto	551	66%	742	63%

Numbers of genes that are significantly up- or down-regulated when comparing the different samples. veg, vegetative mycelium; sex, sexual mycelium; wt proto, wild-type protoperithecia; pro1 proto, protoperithecia from mutant pro1.

^1^The percentage of the up- or down-regulated genes for which a 3’ UTR could be modeled from the RNA-seq data is indicated.

**Figure 3:**
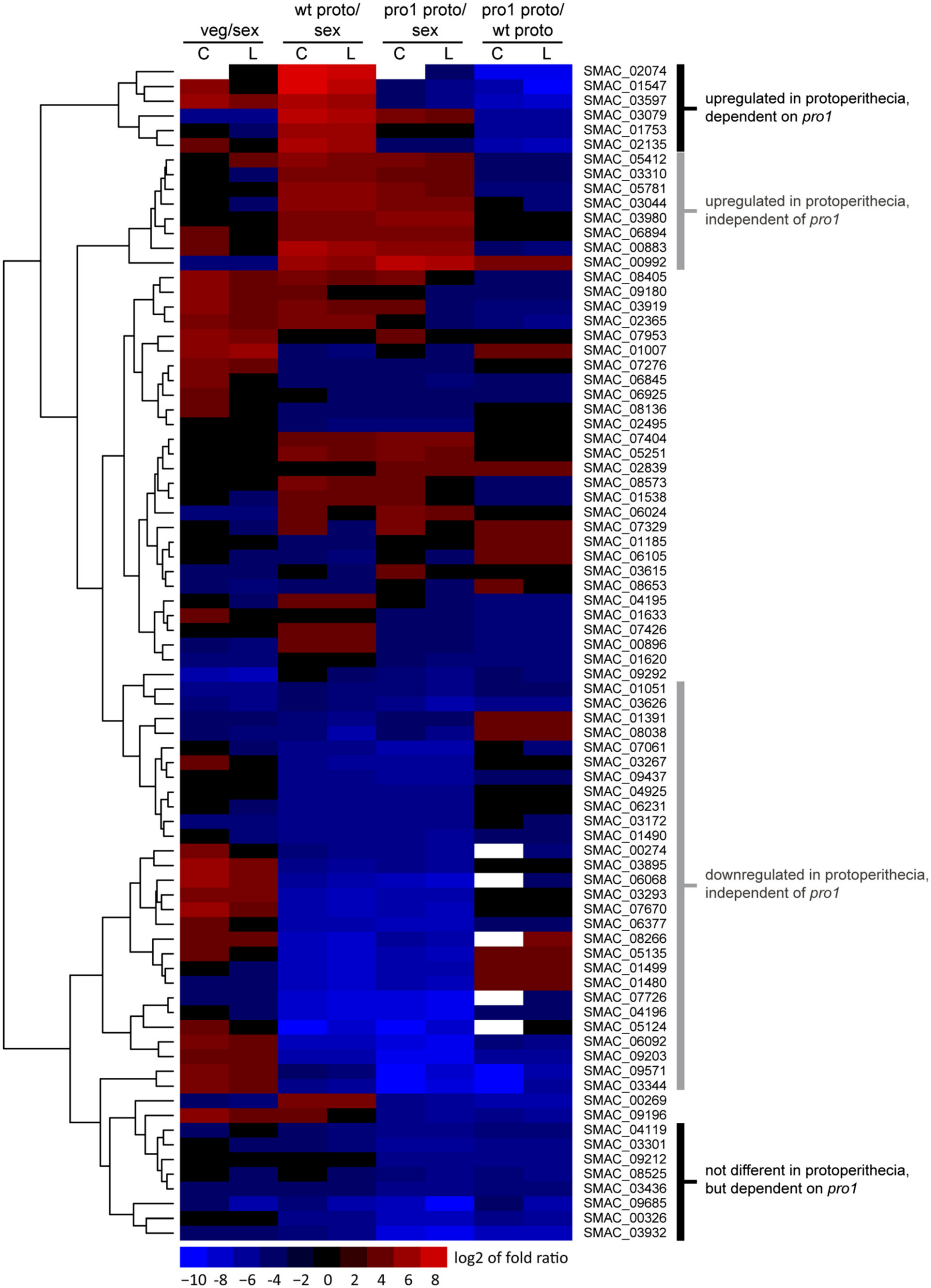
**Expression of putative sugartransporter genes.** Eighty genes contained at least on Sugar_tr domain. Hierarchical clustering and heatmap generation of the log2 of fold ratios as determined by classic (C) and LOX (L) analysis were perfomed in R.

Another hypothesis about fruiting body formation in filamentous fungi assumes that the non-reproductive mycelium first gathers nutrients until a stage of “competence” is reached when the production of fruiting bodies is energetically feasible, and that then the developing fruiting bodies are nurtured by the surrounding non-reproductive mycelium [3,46]. One might speculate that this process could require the transport of massive amounts of nutrients, including carbohydrates. Thus, we analyzed whether the expression of putative sugar transporters changed in the different samples (Figure 3). Of the 80 genes in the *S. macrospora* genome that contain at least on sugar transporter domain, more than 40% were significantly up- or down-regulated in at least one of the conditions investigated, thereby supporting the hypothesis that fruiting body morphogenesis is accompanied by a massive redistribution of nutrients. 

### Analysis of pheromone gene expression in protoperithecia

**Figure 4:**
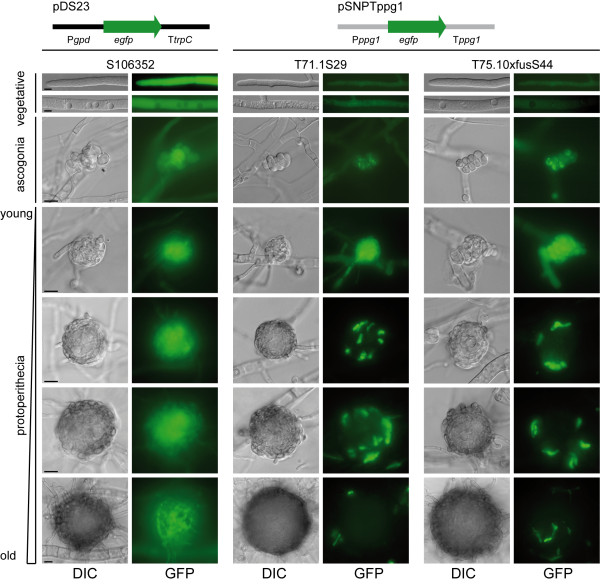
**Microscopic analysis of *egfp*expression under the control of *ppg1 *regulatory regions**. Plasmids pDS23 and pSNPTppg1 were transformed into the *S. macrospora* wild-type. Strain S106352 expresses *egfp* under the control of the *Aspergillus nidulans gpd* promoter and *trpC* terminator from pDS23 (black). Strains T71.1S29 and T75.10xfusS44 express *egfp* under the control of the *S. macrospora ppg1* promoter and terminator regions from pSNPTppg1. Note that EGFP fluorescence under the control of *ppg1* regulatory regions in protoperithecia was observed through a 6% neutral density filter because of very strong fluorescence, which is consistent with high *ppg1* expression in these structures. GFP images are z projections of stacks spanning whole protoperithecia with a plane distance of 0.5 μm. DIC images show the middle plane of the corresponding stack. Scale bar, 10 μm.

We also analyzed if genes that were previously shown to be essential for perithecial development in *S. macrospora* or significantly upregulated during fruiting body formation are differentially regulated in protoperithecia compared to total mycelium ( Additional file 1 Figure S8). Interestingly, we found that both pheromone precursor genes *ppg1* and *ppg2* are strongly upregulated in protoperithecia compared to sexual or vegetative mycelium. The pheromones are required for full fertility [47]; however, where or when they act during the developmental cycle is not yet clear because *S. macrospora* is self-fertile (homothallic), and no obvious fertilization event that requires recognition of compatible partners by pheromones is necessary [27]. To address this question in more detail, we analyzed the expression of an *egfp* reporter gene under the control of the *ppg1* upstream and downstream regulatory regions (Figure 4). No expression was observed in vegetative hyphae, in contrast to the expression of *egfp* from a control vector under the constitutive *gpd* promoter and *trpC* terminator from *A. nidulans*. EGFP fluorescence started to occur in ascogonia (female gametangia) and was strongest in young protoperithecia (diameter ≤ 30 μm). Interestingly, older protoperithecia (> 30 μm) exhibited a distinctly patchy expression pattern in the hyphae of the outer layers, whereas expression of the control vector led to a uniform fluorescence of protoperithecia. On the one hand, these data confirm the transcriptional up-regulation of *ppg1* in protoperithecia as indicated by the RNA-seq analysis; and on the other hand, the microscopic analysis revealed a distinct expression pattern of *ppg1* within protoperithecia. 

### Analysis of *pro1*-dependent gene expression in protoperithecia

**Figure 5:**
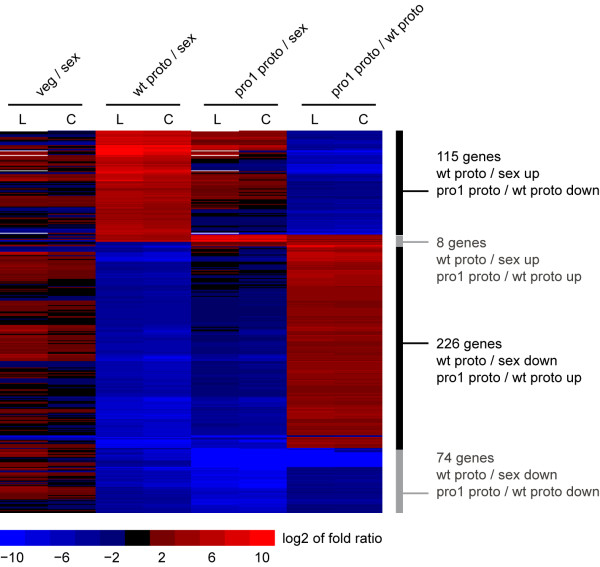
**Genes that are differentially regulated during development and dependent on *pro1*for correct expression.** Hierarchical clustering of the log_2_ of fold ratios as determined by classic (C) and LOX (L) analysis. Log_2_ ratios < −10 or > 10 were set to −10 and 10, respectively, for better scaling visibility. Hierarchical clustering and heatmap generation were performed in R.

To determine which genes are directly or indirectly under the control of transcription factor PRO1 in developing protoperithecia, we looked in more detail at genes that are differentially regulated in wild-type protoperithecia compared to sexual mycelium and are also differentially regulated in pro1 protoperithecia compared to wild-type protoperithecia (Figure 5). This group contains a total of 423 genes, the majority of which are either upregulated in wild-type protoperithecia compared to sexual mycelium and downregulated in pro1 protoperithecia (115 genes) or the other way round (226 genes). For these genes, *pro1* acts as an activator or repressor, respectively, during fruiting body formation. Only eight genes were up-regulated in wild-type protoperithecia compared to sexual mycelium and also up-regulated in pro1 protoperithecia compared to wild-type protoperithecia. Interestingly, six of these eight genes encode proteins that are predicted to be extracellular, including the pheromone genes *ppg1* and *ppg2* (Table 4). We already identified *ppg1* and *ppg2* as being up-regulated in sexual mycelium of mutant pro1 compared to the wild-type in a previous microarray analysis [10]; however, the spatial dimension of this differential expression was not yet known, and the combination of LM and RNA-seq now shows that protoperithecia-specific pheromone gene expression is regulated by the transcription factor gene *pro1*. Two other genes that are up-regulated in both comparisons are homologous to loosenin from the basidiomycete *Bjerkandera adusta* and fasciclin-like protein MoFLP1 from *Magnaporthe grisea*[48,49]. Both proteins have been implicated in cell-wall biogenesis/reorganization, and it is tempting to speculate that the corresponding *S. macrospora* proteins are involved in shaping the outer layers (perithecial wall) of the developing perithecium. 

**Table 4:** Genes up-regulated in wt proto/sex and pro1 proto/wt proto are predicted to encode secreted proteins

**locus_tag**	**best Blast hit withknown function**	**Blast2GO description**	**predicted localization**
SMAC_02071	--		extracellular
SMAC_03636	loosenin (*Bjerkandera adusta*)	riboflavin aldehyde-forming enzyme	extracellular
SMAC_05143	--		nuclear
SMAC_05496	--		extracellular
SMAC_05710	MoFLP1 (*M. grisea*)	fasciclin	extracellular
SMAC_05970	*ppg1*	pheromone precursor	extracellular
SMAC_09568	--		cytosolic
SMAC_12697	*ppg2*	pheromone precursor	extracellular

Putative functions were predicted with Blast2GO [76], putative subcellular localizations with WoLFPsort [77]

We also looked at genes that are physically clustered within the genome, differentially regulated in wild-type protoperithecia, and dependent on *pro1* for correct expression in protoperithecia. Physical clustering of co-regulated genes is often found in fungi for genes involved in secondary metabolism, and it can be used as a tool for identifying novel secondary metabolism pathways [50-53]. We found only two instances of clusters that were differentially regulated in wild-type and pro1 protoperithecia. One cluster comprised putative polyketide synthase genes that were described previously and were most likely acquired by horizontal gene transfer [26]. This cluster was down-regulated in wild-type protoperithecia compared to sexual mycelium and up-regulated in pro1 protoperithecia (data not shown). The second group of clustered genes (*SMAC_09002* to *SMAC_09009*) has the opposite expression pattern, namely up-regulated in wild-type protoperithecia compared to sexual mycelium and down-regulated in pro1 protoperithecia. This cluster does not contain a polyketide or non-ribosomal peptide synthase typical for the corresponding gene clusters; however, one unifying theme of this cluster is that three of its genes encode proteins with the predicted domain of unknown function DUF3328 ( Additional file 1 Figure S9). Whether genes from this family play a role in fruiting body formation remains to be elucidated.

**Figure 6:**
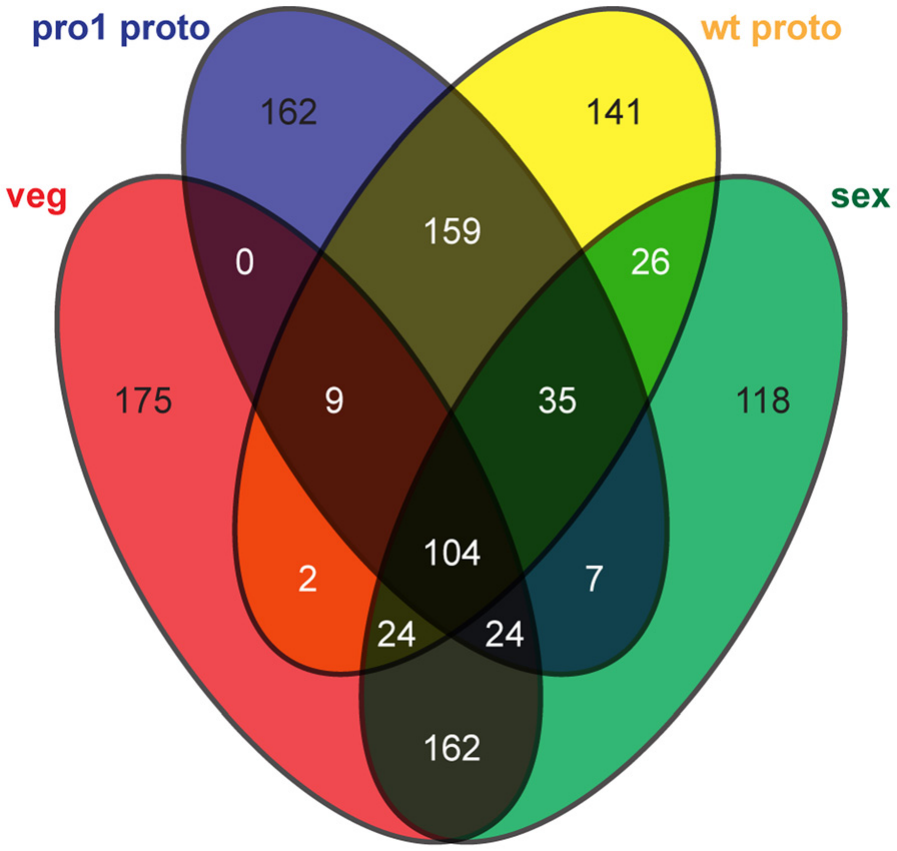
**Venn diagram of geneswith top 500 readcounts for each sample**. Numbers of genes that are in the top 500 group for one or more or the four samples (vegetative mycelium, sexual mycelium, wild-type protoperithecia, pro1 protoperithecia) are given. In this analysis, only reads that map within 100 to 400 bases from the 3’ end of the mRNA were used to account for the 3’ bias in the microdissection samples and different mRNA lengths. An analysis using read counts for complete predicted mRNAs gave similar results (data not shown).

Next, we investigated the transcripts that were most abundant in protoperithecia from the wild-type and mutant pro1, and whether there was a difference to the most abundant transcripts in sexual or vegetative mycelium. For this analysis, we counted reads that mapped to the 3’ end (100–400 nt from the 3’ end) of each predicted mRNA. This approach was chosen to account for the 3’ bias in the microdissected samples, and it generates numbers that are largely independent of transcript length. Read counts were normalized to the total number of counted reads in each sample, and the average read count from the two independent repetitions of each sample was used to determine the 500 genes in each of the four samples that had the highest number of reads (Figure 6, Additional file 3). The analysis showed that 104 genes were present in the top 500 in all four samples, and that sexual mycelium and vegetative mycelium, and protoperithecia from wild-type and pro1 had overlaps of 162 and 159 genes, respectively. In contrast, the number of common genes among the top 500 from the mycelial samples and the protoperithecial samples was much lower (Figure 6). This again indicates that the transcriptional landscapes of non-reproductive mycelia versus protoperithecia are rather different, and that overall transcription in sexual mycelium is driven by the non-reproductive hyphae that make up the majority of this sample.

**Figure 7:**
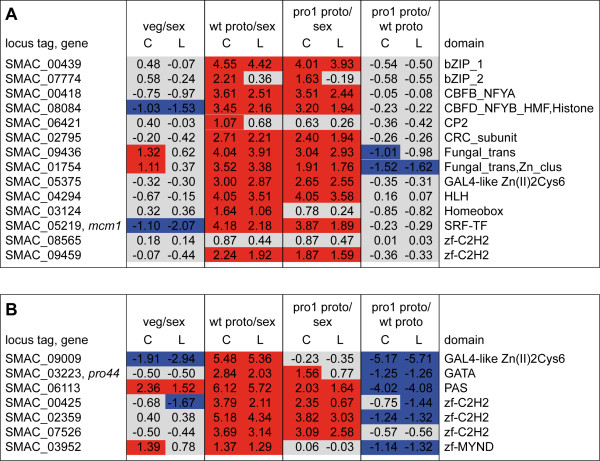
**Expression ratios for transcriptionfactors among the geneswith top 500 readcounts.** (**A**) Expression ratios for the transcription factors among the 500 genes with the highest number of read counts in wild-type and mutant pro1 protoperithecia. Expression of these genes is largely independent of *pro1*. (**B**) Expression ratios for the transcription factors among the 500 genes with the highest number of read counts in wild-type protoperithecia but not pro1 protoperithecia. These genes are most likely dependent on *pro1* for correct expression. Expression ratios in (**A**) and (**B**) are given as log_2_ values, and log_2_ ratios >1 and <−1 are indicated in red and blue, respectively. The genes in (**A**) are mostly not differentially expressed in pro1 protoperithecia compared to wild-type protoperithecia (indicated by the grey coloring), whereas the genes in (**B**) have a tendency towards down-regulation in pro1 protoperithecia, as expected for genes that are dependent on *pro1* for correct expression. Protein domains were predicted with HMMER using the Hidden Markov models from the pfam database [74,75].

We specifically analyzed whether any transcription factor genes are only present among the top 500 genes in wild-type protoperithecia or in wild-type and pro1 protoperithecia, but not in the mycelial samples. We found 14 putative transcription factors among the top 500 genes in both wild-type and mutant protoperithecia, and seven putative transcription factors among the top 500 genes in wild-type, but not pro1 protoperithecia (Figure 7). Analysis of the gene expression ratios showed that the first group of transcription factors is largely independent of *pro1* (no difference in expression between pro1 and wild-type protoperithecia, Figure 7A), whereas the second group depends on *pro1* for upregulation in protoperithecia (genes down-regulated in pro1 compared to wild-type protoperithecia, Figure 7B). Transcription factors that are strongly expressed in protoperithecia might be involved in regulating the expression of downstream genes that mediate fruiting body morphogenesis. Two of these transcription factors were already shown to be essential for fruiting body formation, namely *mcm1* and *pro44*. Mutations in *mcm1* or *pro44* lead to sterility, and the corresponding mutants are able to produce protoperithecia, but not mature perithecia [28,54]. A comparison with homologous transcription factors from *N. crassa* and *F. graminearum* revealed that, out of the 21 transcription factors, knockout strains have been analyzed for 12 and 19 genes from *N. crassa* and *F. graminearum*, respectively, in large-scale knockout projects with these two organisms [55,56]. Of these deletion mutants, three showed defects in sexual development in *N. crassa*, and 12 in *F. graminearum* ( Additional file 1 Table S1). Homologs of *pro44* were sterile in both species, and the corresponding homolog of *Aspergillus nidulans* was also shown to be essential for sexual development [57]. Thus, the transcription factors from this analysis might be promising candidates for further functional studies, especially those with developmental phenotypes in other filamentous fungi. 

## Discussion

Fungal fruiting body formation is a complex process that requires coordinated patterns of gene expression in time and space. Even though a number of genes that are essential for this process have been isolated from several model organisms, no unifying theory yet explains the spatio-temporal succession of developmental events leading to the mature fruiting body [3]. One way to learn more about the genes that are active during this process is to look at genome-wide expression patterns at different developmental stages, but this is difficult in many ascomycetes, because fruiting bodies are often rather small (< 500 μm for the mature fruiting body) and difficult to separate from surrounding, non-reproductive hyphae. In the present study, we used LM and RNA-seq to analyze gene expression in protoperithecia from the model organism *S. macrospora*. To the best of our knowledge, this study is the first time that a combination of these methods has been used for the analysis of fungal gene expression. Our study demonstrates that young fruiting bodies of *S. macrospora* can be isolated using LM, and RNA extracted from these samples in sufficient amounts for RNA-seq after two rounds of linear amplification. The amplification process largely conserves expression ratios, as demonstrated by comparing the expression of selected genes prior to amplification with the RNA-seq results, which is consistent with results from other organisms where linear RNA amplification was used to prepare samples for microarray hybridization [19,20]. We also found some overlap with prior microarray experiments in which we compared gene expression in vegetative and sexual mycelia; however, in these experiments we used mycelia grown in defined medium [9], whereas for RNA-seq analysis, RNA from mycelia grown in defined medium and cornmeal medium were pooled. Therefore, some differences between these experiments might be due to different growth conditions ( Additional file 1 Table S2). Furthermore, we demonstrated that the regulatory regions of *ppg1* can drive expression of an *egfp* reporter gene in protoperithecia, as predicted by the RNA-seq analysis. In addition, fluorescence microscopy analysis revealed distinct expression patterns for *ppg1* in the outer layers of the protoperithecium. This finding might be consistent with a hypothesis that has been put forward for *N. crassa* that predicts that pheromones are not only signaling molecules that enable the recognition of mating partners, but that they also play a role in the attachment (“conglutination”) of hyphae forming the rigid outer perithecial wall [58]. A role for pheromones as “molecular glue” might explain the expression of *ppg1* in cells of the protoperithecial outer layers in *S. macrospora*.

In a study of gene expression in several tissues of different metazoans, Hebenstreit et al. found that genes can be grouped into two classes, namely genes with high and low expression, independent of tissue type, species or type of experiment (microarray analysis or RNA-seq) [59]. This classification resulted in two distinct peaks when plotting the distribution of gene expression levels. We wondered whether this distribution might also be found in fungi, but plots of the distribution of gene expression levels showed different patterns for our data ( Additional file 1 Figure S10, Additional file 4). We observed a single main peak in both vegetative and sexual mycelium, whereas the frequency distribution in wild-type and pro1 protoperithecia could be dissected into three peaks. This difference indicates that, in contrast to metazoans, fungal genes might not generally fall into two main classes of expression. One reason might be that in the case of sexual and vegetative mycelium, pooled RNA samples were used from mycelia grown in different types of media. These mycelia might express different sets of genes at high and low levels, and such a mixture would drive overall expression frequencies towards intermediate values [59], resulting in a single peak as observed. However, the multiple peaks for protoperithecia cannot be explained by a mixture of different samples. The analysis by Hebenstreit indicated that the genes from the high expression group constitute the active and functional transcriptome of the cell, whereas the genes from the low expression group show “leaky” expression [59]. Our data indicate that the situation in fungi might be different, but further analysis will be needed to clarify this point.

In previous studies, we used cross-species microarray hybridization to hybridize *S. macrospora* targets on *N. crassa* cDNA or oligonucleotide microarrays [8-11]; however, in these analyses, less than 50% of all genes on the arrays gave a significant signal. The use of RNA-seq dramatically improves detection levels, with more than 90% of all genes being detected in at least one of the sequenced samples. Also, the comparison of gene expression revealed that the overall expression in sexual mycelium is more similar to that of vegetative mycelium than protoperithecia. This finding indicates that gene expression in the sexual mycelium is most likely driven to a large extent by genes expressed in the non-reproductive hyphae making up the bulk of the mycelium and that, in order to study genes specifically expressed in developing fruiting bodies, the microdissection method applied here provides a much better spatial resolution and a much more detailed and specific picture of gene expression during development. Especially weakly expressed, fruiting body-specific genes would most likely not be detected as differentially expressed (or at all) in an expression study using only sexual mycelium. Previous approaches for isolating fruiting bodies for gene expression studies were performed in *N. crassa* and *F. graminearum*, using EST sequencing with RNA from mature fruiting bodies [5], or by analyzing different stages of fruiting bodies by microarray hybridization [6,7,13]. The analysis by Hallen et al. [7] was performed with Affymetrix GeneChips for *F. graminearum*, and signals were detected for nearly 80% of all transcripts, whereas the EST analysis was limited by a comparatively low sequencing depth, and in the other two microarray studies [6,13], only 10% of all genes gave signals or were represented on the arrays. In all studies, fruiting bodies were harvested by scraping developing structures from a plate, and these preparations might contain an undetermined amount of non-fruiting body mycelia, especially in the early stages of development when fruiting body precursors are small. Therefore, the present analysis of microdissected protoperithecia allowed the analysis of gene expression solely in these structures for the first time. An additional advantage of RNA-seq is that the data can be used also for annotation purposes and, in the case of *S. macrospora*, allowed the modeling of more than 50% of the UTRs, and the improvement of exon-intron structures for about 1,000 genes (~ 10% of the predicted genes in the genome).

The analysis of gene expression ratios and the 500 genes with the highest number of reads in each of the four sequenced samples showed that expression in protoperithecia from the wild-type and mutant pro1 is more similar to each other than to either vegetative or sexual mycelium, indicating that the transcriptional landscape of protoperithecia is distinct from that of non-reproductive mycelium. However, there are also significant differences between protoperithecia from the wild-type and the sterile mutant pro1 that can form protoperithecia, but not mature fruiting bodies. More than 400 genes were significantly up- or downregulated in pro1 protoperithecia compared to wild-type protoperithecia, and therefore might be direct or indirect targets of PRO1. Among the genes that are dependent on *pro1* for correct expression in protoperithecia are the pheromone precursor genes, several genes that might be involved in perithecial wall morphogenesis, and a number of transcription factors. Previous analyses identified several mutants in which the pheromone precursor genes are differentially regulated in sexual mycelium compared to the wild-type [8,10,11,60]; however, no information was available about the spatial regulation of the expression of developmental genes prior to this study. One might hypothesize that *pro1* is involved in balancing the expression of genes involved in the formation of the rigid perithecial wall because the pheromone precursor genes and several other genes predicted to be involved in cell-wall biosynthesis are up-regulated in pro1 protoperithecia (Table 4).

## Conclusions

We have established a combined LM/RNA-seq approach to analyze gene expression in developing fungal sexual structures, and used it to analyze the transcriptome of young fruiting bodies in the wild-type and the sterile mutant pro1 of *S. macrospora*. *pro1*, which encodes a transcription factor, is essential for sexual development. To the best of our knowledge, this is the first genome-wide analysis of genes that are dependent on a development-specific transcription factor for correct expression in a defined developmental structure in fungi. Genes that are differentially expressed in protoperithecia are prime candidates for further functional analysis to unravel the spatio-temporal sequence of events leading to the mature fungal fruiting body. Together with three recent studies in Arabidopsis and tomato, as well as rat neurons [32-34], our analysis of fungal development demonstrates the power of a combined approach of LM and RNA-seq to analyze cell type-specific or tissue-specific gene expression in complex, multicellular structures.

## Methods

### Strains and culture conditions

*S. macrospora* strains used in this study were the wild-type (FGSC 10222, [26]) and the sterile mutant pro1 [36] from the culture collection of the Department of General and Molecular Botany. For propagation, strains were grown on cornmeal medium as described previously [61]. For RNA extraction from vegetative or sexual mycelium, strains were grown in liquid medium as surface cultures (sexual mycelium) or submerged (vegetative mycelium) in cornmeal medium or defined medium as described previously [10,41].

### Laser microdissection

Laser microdissection was performed with a CellCut Plus system (MMI, Molecular Machines and Industries, Zürich, Switzerland) comprising an Olympus IX81 inverted microscope equipped with a UV laser (355 nm), microscope stage and isolation cup holder. For microdissection, strains were first grown on cellophane-coated cornmeal agar plates for 3 days. Hyphae from these cultures were used to inoculate MMI membrane slides coated with a thin layer of medium (150–200 μl of cornmeal medium with 1% agar). Slides were incubated in a glass Petri dish with approximately 5 ml of water to prevent the samples from drying. Strains were grown for 4–6 days at 25°C in constant light. For fixation, acetone and ethanol gave similar results in RNA extraction and qRT-PCR analysis (data not shown); in the experiments described here, slides were fixed in ethanol at 4°C over night. Prior to microdissection, the slides were air-dried for 30 min, then the mycelium-bearing side was covered with a glass microscope slide and the sample was inserted into the microscope stage with the membrane slide on top (Figure 1). Protoperithecia (~20 μm in diameter) were labeled manually using the MMI CellTools software and cut with the UV laser in a distance of ~2-5 μm from the edge of the protoperithecium. This distance minimizes laser damage to the protoperithecium, and ensures that only minimal amounts of unrelated hyphae were isolated. Approximately 100–300 protoperithecia were collected from each slide using an MMI isolation cup (Figure 1).

### RNA preparation, RNA amplification, and qRT-PCR

RNA was isolated from microdissected protoperithecia using the Arcturus PicoPure kit (Applied Biosystems, Carlsbad, CA, USA) according to the manufacturer’s protocol with the following modifications: 50 μl extraction buffer was pipetted into the isolation cup which was then inverted to cover the cap with the attached microdissected samples and incubated at 42°C for 30 min. Isolation cups were centrifuged (5 min, 800 g) to collect the samples, and then the following procedure was performed three times: cups were frozen in liquid nitrogen for 30 s and then vortexed at room temperature for 30 s. Afterwards, the solution was thawed at 42°C; then extraction was performed using the PicoPure columns according to the manufacturer’s protocol, including a 15 min DNase incubation step (RNase-free DNase Set, Qiagen, Hilden, Germany) after the first washing step. RNA was eluted in 20 μl water. Reverse transcription and qRT-PCR of RNAs isolated from microdissected samples was performed as described previously [10,41], but instead of 1 μg of RNA, the complete 20 μl of eluted RNA was used for reverse transcription. Primers for qRT-PCR are given in Additional file 1 Table S3. Amplification of RNA from microdissected samples was performed with the TargetAmp 2-round aRNA amplification kit 2.0 (Epicentre Biotechnologies, Madison, WI, USA) according to the manufacturer’s protocol with the following modifications. In the second amplification round, first strand cDNA synthesis was performed using primer T7N9 and second strand synthesis using primer oligo-dT(24)-anchored ( Additional file 1 Table S3) instead of the primers supplied with the kit in order to obtain polyA-tailed aRNA that could be used directly for Illumina/Solexa library preparation with the same protocol used for total RNA from non-microdissected samples. For vegetative and sexual mycelium, RNA preparation, reverse transcription and qRT-PCR were performed as described previously [10,41,62].

### Illumina/Solexa sequencing by synthesis and cleaning of primary sequence data

Five micrograms of amplified RNA from microdissected sample was used for Illumina/Solexa sequencing. For sexual and vegetative mycelium, RNA concentrations were quantified photometrically, and equal amounts (50 μg per condition) of RNA from the following growth conditions (independent biological replicates for each growth condition) were pooled: growth on cornmeal medium for 3d, 4d, and 5d, growth on defined medium for 3d, 4d, and 5d; surface cultures for sexual mycelium and shaken cultures for vegetative mycelium. cDNA preparation and Illumina/Solexa sequencing were performed at GATC Biotech (Konstanz, Germany). cDNA preparation was performed using the SMART cDNA library construction kit (Clontech, Mountain View, CA, USA) based on oligo-dT priming for first strand synthesis. For each sample, two independent biological replicates were analyzed (single reads of 35 to 101 bases), each was sequenced in one lane of the GAII (samples SM1 and SM2) or HiSeq 2000 (all other samples, for overview of read numbers, see Table 1). Raw sequence data were analyzed and trimmed with custom-made Perl scripts (available at http://c4-1-8.serverhosting.rub.de/public/software.html). Sequence reads that contained undetermined bases (“N”) were removed. The remaining reads were checked for base quality from the 3’ end, and bases with a quality score of less than 10 (standard Sanger phred scores, [63]) were removed consecutively; reads longer than 20 bases after 3’ trimming were kept for mapping.

### Mapping of RNA-seq reads, UTR predictions, and improvement of annotations

The cleaned sequence reads were mapped to the *S. macrospora* reference genome [26] using Tophat [42]. UTRs were predicted according to the principle shown in Additional file 1 Figure S2 with search algorithms described in Additional file 1 Method S1. The annotation of predicted open reading frames was checked and improved based on the RNA-seq data using custom-made Perl scripts to implement the algorithm shown in Additional file 1 Figure S3 and Additional file 1 Method S2. Novel open reading frames were annotated manually based on confirmed splice reads outside of predicted genes. The improved annotation of the *S. macrospora* genome is available from the ENA database under accession numbers CABT02000001-CABT02001583 and from http://c4-1-8.serverhosting.rub.de/public/.

### Quantitative analysis of gene expression based on RNA-seq data

Custom-made Perl scripts were used to determine the number of reads that mapped to each annotated protein-coding gene based on the SAM files with the mapping information (output from Tophat [42]) and using the algorithm shown in Additional file 1 Figure S4. Reads were counted stringently in that only reads (> 34 bases) were counted where both ends mapped to the same annotated feature. This approach leads to some loss of reads that map with one end to the UTR of a gene where UTRs are not yet annotated; however, the more stringent counting also prevents spuriously mapped reads from being counted. Raw read counts were used for quantitative analysis with four different methods. The first two approaches were with the Bioconductor packages DESeq [64] and baySeq [65] in the R computing environment (version 2.12.1). However, differential expression estimated by these methods was not in good agreement with previous results from other techniques (microarray, qRT-PCR, and Northern blot, data not shown), most likely due to the fact that the statistical models upon which these methods are based are only valid if the majority of genes (~ 90%) are not differentially expressed. However, in some of our samples, considerably more genes are differentially expressed. Therefore, we employed two other methods to calculate gene expression ratios. One method was based on the LOX program that calculates expression ratios and Bayesian credible intervals and P-values for differential expression [45]. The other method (called “classical analysis”) consists of the calculation of expression ratios, standard deviation, and coefficient of variance from read counts normalized to the total number of read counts for the sample, similar to what was described previously for microarray analyses [10]. In the classical analysis, genes were sorted into five groups (0–4) according to the following criteria: genes in group 4 have ratios of ≤ 0.25 or ≥ 4 in all independent biological replicates, genes in group 3 have a mean ratio of ≤ 0.25 or ≥ 4 and a coefficient of variance < 0.5, genes in group 2 have ratios of ≤ 0.5 or ≥ 2 in all independent biological replicates, genes in group 1 have a mean ratio of ≤ 0.5 or ≥ 2 and a coefficient of variance < 0.5, and group 0 contains all other genes (with the exception of genes for which no ratios could be calculated due to a lack of read coverage, these were not included in the analysis). Overall, results from both methods agreed better with previous results and additional qRT-PCR analyses than results from DESeq or baySeq. To classify genes as differentially expressed, a consensus was determined for each gene based on the results from both the classical and LOX analysis; a gene was labeled as up-regulated (1), down-regulated (−1) or not differentially expressed under the conditions that were compared, when the following criteria were met: (a) for a gene to be classified as differentially regulated in the comparison veg/sex (vegetative mycelium versus sexual mycelium) or pro1 proto/wt proto (pro1 protoperithecia versus wild-type protoperithecia), expression ratios from both classical and LOX analysis had to be > 4 and < 0.25, LOX Bayesian probability for differential expression = 1, and the gene had to be in groups 1–4 in the classical analysis; (b) for a gene to be classified as differentially regulated in the comparison wt proto/sex (wild-type protoperithecia versus sexual mycelium) or pro1 proto/sex (pro1 protoperithecia versus sexual mycelium), expression ratios from both classical and LOX analysis had to be > 8 and < 0.125, LOX Bayesian probability for differential expression = 1, and the gene had to be in groups 1–4 in the classical analysis. We used two different thresholds for differential expression depending on the conditions that were compared, because our analyses showed that lower cutoffs in comparisons of RNA-seq data from microdissected samples and non-microdissected samples resulted in relatively large numbers of false-positives, therefore a more stringent cutoff was used. Results from the classical and LOX analysis as well as the consensus analysis are given in Additional file 2. For the analysis of reads that mapped to different genomic regions (e.g., exons, introns, intergenic regions), reads were counted based on the SAM files with the mapping information using custom-made Perl scripts as described above. To determine the distribution of expression frequencies, the coverage for locus tags of protein-coding gene was determined as the average coverage for the bases of the predicted mRNA (normalized to coverage per kilobase per million counted bases in the sample, Additional file 4). Curve fitting and clustering of the data by expectation-maximization was performed on the log_2_-transformed RNA-seq data using the R package mclust [66].

### Phylogenetic analysis

Multiple alignments were created in CLUSTALX [67] and trimmed with Jalview [68], and the same alignment was used for analysis by neighbor joining (NJ) and maximum parsimony (MP). Phylogenetic analyses were performed with PAUP version 4.0b10 for Windows (D.L. Swofford, distributed by Sinauer Associates, copyright 2001 Smithsonian Institution) for NJ and MP analyses using 10,000 bootstrap replicates [69]. Consensus trees were graphically displayed with TREEVIEW [70].

### Cloning procedures and transformation of *S. macrospora*

Plasmid pSNPTppg1 containing *egfp* flanked by the upstream and downstream sequences (1 kb each) of *ppg1* was generated by homologous recombination in yeast as described by Colot et al. [55] based on plasmid pRSnat which contains a nourseothricin resistance cassette for selection in *S. macrospora*[8]. *S. macrospora* was transformed as described previously using a combination of Glucanex 100 G (Novozymes A/S, Bagsvaerd, Denmark) and 1.4 U/ml chitinase (ASA Spezialenzyme GmbH) for protoplast generation [28,71].

### Fluorescence microscopy

For fluorescence microscopy of transformants carrying *egfp* expression plasmids, strains were grown on glass slides with a thin layer of cornmeal medium and analyzed with an AxioImager fluorescence microscope as described previously [72,73]. EGFP fluorescence under the control of *ppg1* regulatory regions in protoperithecia was observed through a 6% neutral density filter because of very strong fluorescence. All other observations were made without neutral density filters.

### Accession numbers

The improved version 2 of the *S. macrospora* genome generated during this work was submitted to the ENA database and is available under the accession numbers CABT02000001-CABT02001583. The RNA-seq reads and results of expression quantification generated in this project were submitted to the GEO database (accession number GSE33668).

## Competing interests

The authors declare that they have no competing interests.

## Authors’ contributions

IT and MN carried out the molecular genetics experiments, IT and GW performed laser microdissection, IT carried out fluorescence microscopy, MN performed the bioinformatics analyses, MN drafted the manuscript, IT and UK helped draft the manuscript, and MN and UK designed the study. All authors read and approved the final manuscript.

## Supplementary Material

Additional file 1 **Figure S1. Read coverage and UTR predictions.** Figure S2. Algorithm for modelling UTRs. Figure S3. Algorithm for improving exon-intron structures based on RNA-seq data. Figure S4. Algorithm for counting reads that map to predicted features (e.g. mRNAs) for implementation in Perl. Figure S5. Analysis of the genome-wide coverage of different genomic regions. Figure S6. Venn diagram of the number of genes without any read counts. Figure S7. Transcript analysis of selected genes using qRT-PCR and RNA-seq. Figure S8. Expression of known developmental genes. Figure S9. Phylogenetic tree of all DUF3328 proteins from *Sordaria macrospora*, *Neurospora crassa* and *Neurospora tetrasperma*. Figure S10. Distribution of gene expression levels. Table S1. Transcription factors among the genes with the top 500 read counts and their homologs in *Neurospora crassa* and *Fusarium graminearum*. Table S2. Comparison of results from RNA-seq and microarray analysis. Table S3. Oligonucleotides used in this study. Method S1. UTR predictions from RNA-seq data. Method S2. Annotation of novel gene models based on RNA-seq data. Click here for file

Additional file 2 Contains normalized read counts and expression analyses for different conditions. Click here for file

Additional file 3 Contains an analysis of the top500 genes with respect to read counts for each of the four conditions. Click here for file

Additional file 4 Contains base counts per locus tag for eight independent RNA-seq experiments. Click here for file
