# Supplementary material for: Combining laser microdissection and RNA-seq to chart the transcriptional landscape of fungal development
Source: BMC Genomics. 2012 Sep 27;13:511. doi: 10.1186/1471-2164-13-511 (PMC3472292; doi:10.1186/1471-2164-13-511)
Supplement: Additional file 1 — Figure S1. Read coverage and UTR predictions. Figure S2. Algorithm for modelling UTRs. Figure S3. Algorithm for improving exon-intron structures based on RNA-seq data. Figure S4. Algorithm for counting reads that map to predicted features (e.g. mRNAs) for implementation in Perl. Figure S5. Analysis of the genome-wide coverage of different genomic regions. Figure S6. Venn diagram of the number of genes without any read counts. Figure S7. Transcript analysis of selected genes using qRT-PCR and RNA-seq. Figure S8. Expression of known developmental genes. Figure S9. Phylogenetic tree of all DUF3328 proteins from Sordaria macrospora, Neurospora crassa and Neurospora tetrasperma. Figure S10. Distribution of gene expression levels. Table S1. Transcription factors among the genes with the top 500 read counts and their homologs in Neurospora crassa and Fusarium graminearum. Table S2. Comparison of results from RNA-seq and microarray analysis. Table S3. Oligonucleotides used in this study. Method S1. UTR predictions from RNA-seq data. Method S2. Annotation of novel gene models based on RNA-seq data. [file 1471-2164-13-511-S1.pdf]

Additional file 1 for Teichert et al.

contains Supplemental Figures S1-S10, Tables S1-S3 and Methods S1-S2

1. Supplemental Figures

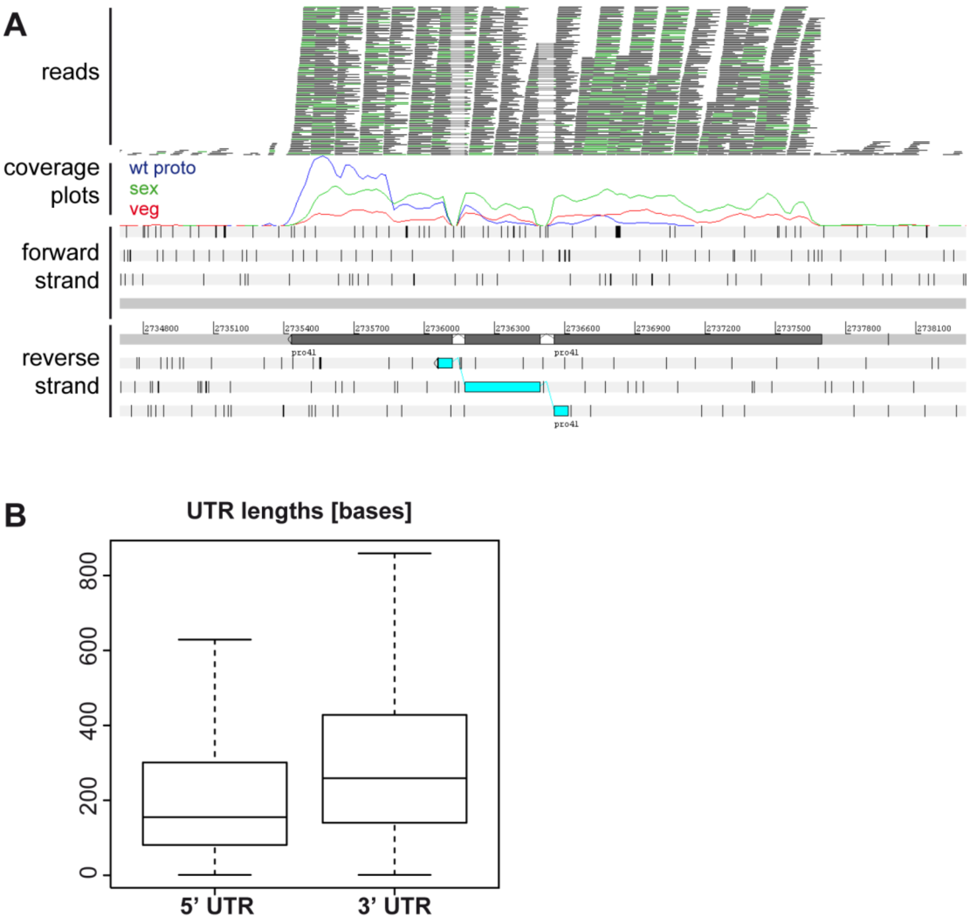

Figure S1. Read coverage and UTR predictions (**A**) RNA-seq read coverage for the *pro41* gene locus. BAM files containing the mapping information were visualized in the Artemis genome browser [43]. The coding region (blue arrow) and the mRNA structure (gray arrow) were determined in previous experiments [9], and RNA-seq read coverage correlates accurately with the mRNA. The coverage plot shows the 3' bias of the microdissection samples (wt proto: blue) whereas in the mycelial samples (veg: red, sex: green), the complete mRNA is covered. The introns are not covered (as expected), but spanned by reads as indicated by light gray lines. (**B**) Length distributions for predicted 5' and 3' UTRs. The box plots show the length distributions of predicted 5' and 3' UTRs with the median value as a horizontal line in the box between the first and third quartiles. Outliers are not included in the graph.

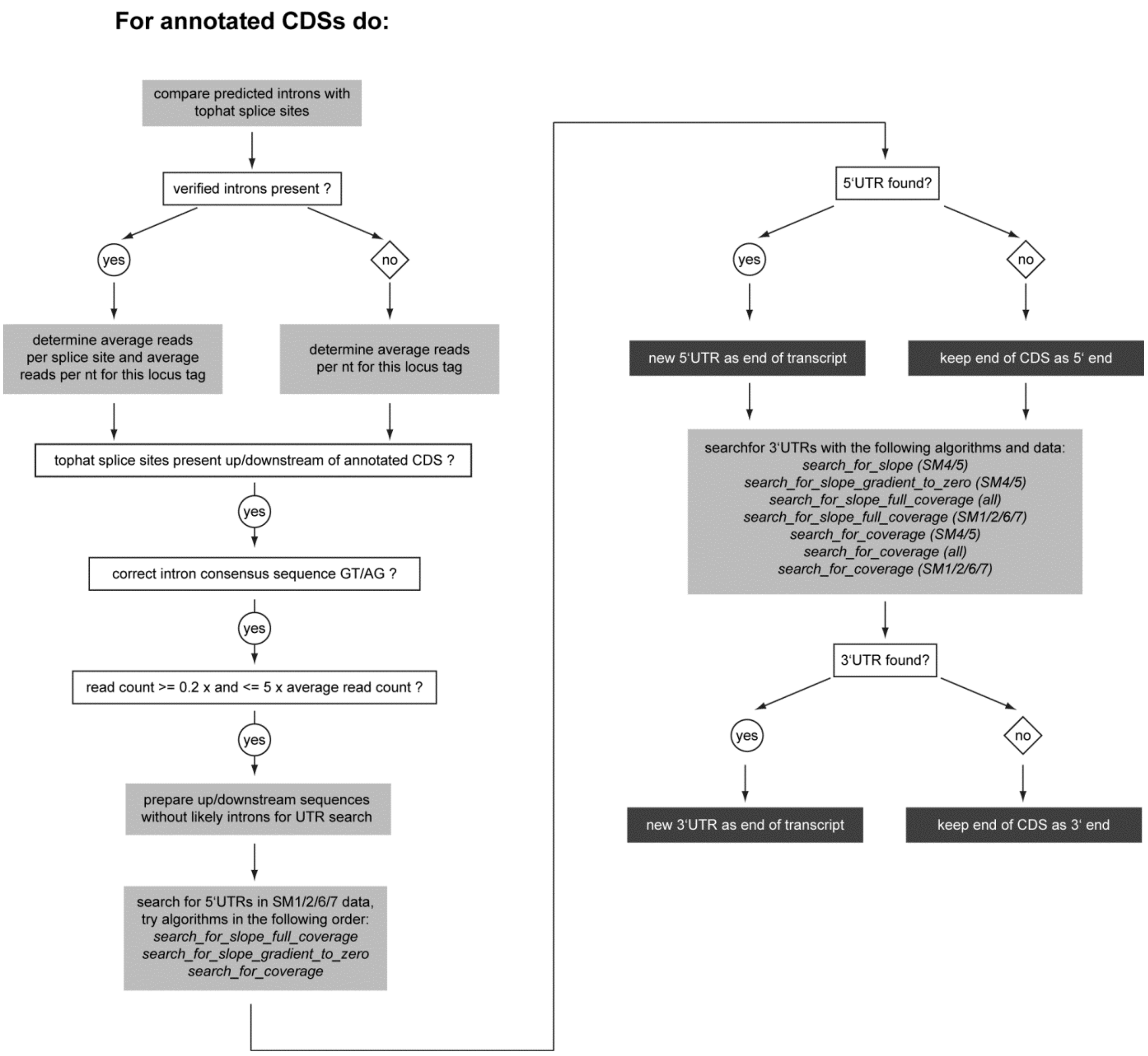

Figure S2. Algorithm for modelling UTRs.

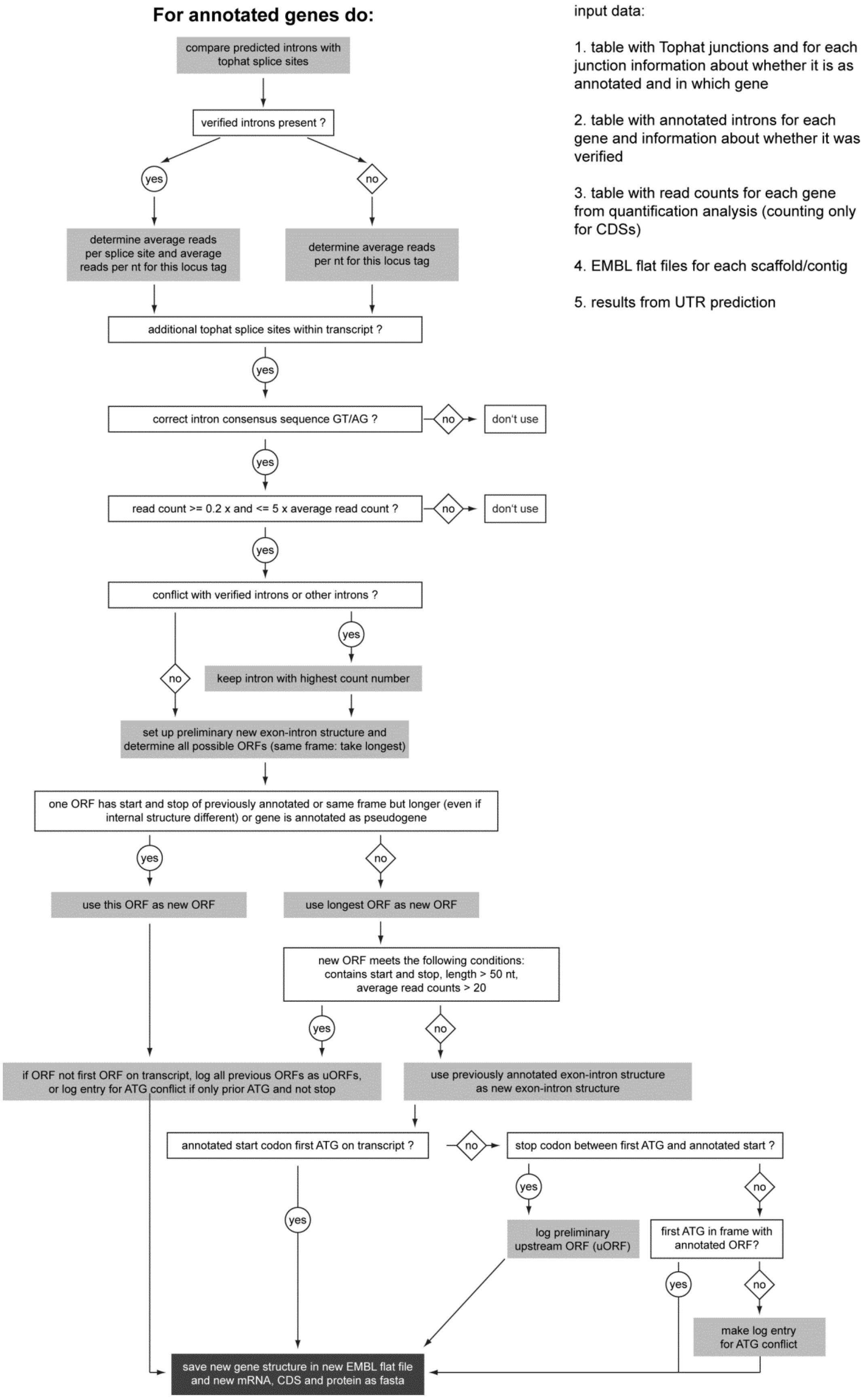

Figure S3. Algorithm for improving exon-intron structures based on RNA-seq data.

A) Prepare genome information from EMBL flat files

Make hash with positions for mRNAs (CDS for mitochondrial genes): key = Scaffold+Position, value = locus_tag+bin

Binning: each mRNA should be divided into four parts starting at the 5' end.

Example: In both cases, bin2 contains only those positions that are covered by exons, not the intron!

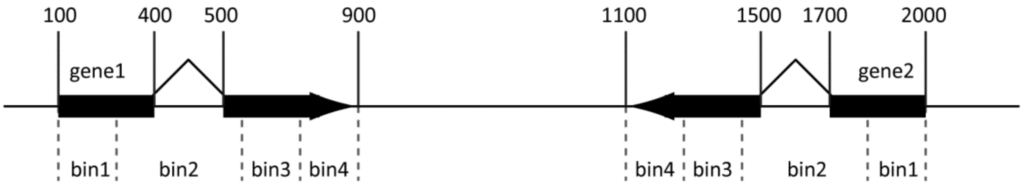

Example for dividing mRNA length in bins:

e.g. mRNA length 102 bases, integer of length(mRNA)/4 = 25

therefore:
bin1: 1-25
bin2: 26-50
bin3: 51-75
bin4: 76-102

Implementation:
1. make array with positions that are covered by mRNA, e.g. 10..40, 70..141 (no strand specificity needed here, comes when moving through array).
2. sort array (numeric upwards for forward strand, numeric downwards for reverse strand).
3. walk through each member of hash, start with bin1, keep counting to bin size, then go to next bin, at the end put rest of mRNA in last bin.

B) Count reads in bins (1-4) if they map to an mRNA

Possible arrangements for mapped reads with respect to annotated genes
(Not shown are reads that don't map to annotated genes or those where split reads map to two different genes or cover >1 intron)

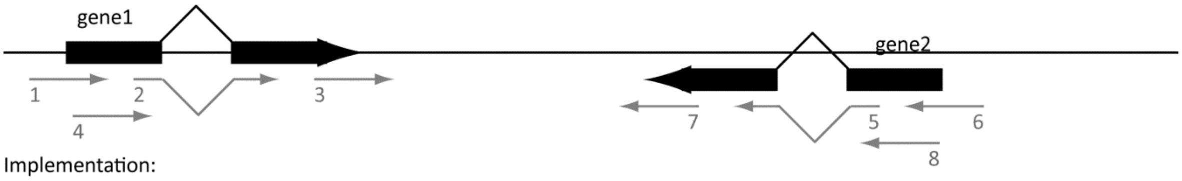

Implementation:
1. take read line from SAM file and determine whether split read or completely mapped to one position (e.g. „36M")
2. if split read: split mapping info and get positions for all parts
3. check for start and end position of read whether read maps to annotated feature
   if yes, check whether start and end map to same feature or one end doesn't map to feature (see figure, reads 1, 3, 6, 7),
   if both ends map (e.g. reads 2, 4, 5, 8 in figure), count read in bin (implemented as hash) with lowest number (e.g. in bin1 if one end maps to bin1 and the other to bin2), if only one end maps, don't count in program „stringent", count in other version.
   Not counting reads that map only with one end leads to loss of reads that map in the UTR regions of genes where UTRs were not annotated, but keeps reads from precursor mRNAs etc. that map in introns out of the count.

C) Make final count for each gene and determine measurement for evenness of coverage

Implementation:
1. sum up counts of all four bins for each gene to get total count
2. for evenness of coverage determine mean count number across bins, standard deviation, coefficient of variance
   and CV/length of mRNA as a meaure for evenness (longer genes tend to have fewer full-length cDNAs)
3. output: all data from points 1 and 2 for each gene

Figure S4. Algorithm for counting reads that map to predicted features (e.g. mRNAs) for implementation in Perl.

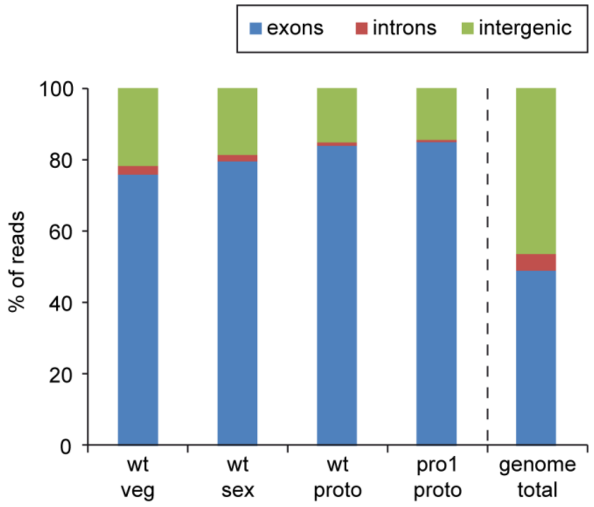

Figure S5. Analysis of genome-wide coverage of different genomic regions (exons of protein-coding genes, introns of protein-coding genes, intergenic regions). Non-coding RNAs and repeats are not included in this analysis. Percent of reads that map to the corresponding regions are shown (only reads are counted where both ends map to the same type of region). At the right end of the graph (separated by a dashed line), the relative distribution of these regions across the genome is indicated.

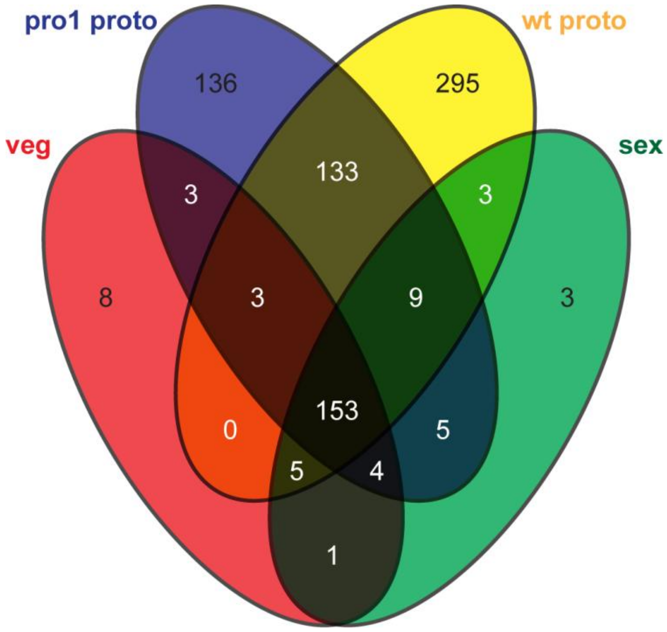

Figure S6. Venn diagram of numbers of genes without any read counts. Read counts were summed up from the two replicates of each condition (veg, sex, wt proto, pro1 proto). In total, there are 764 genes that have no counts in at least one condition.

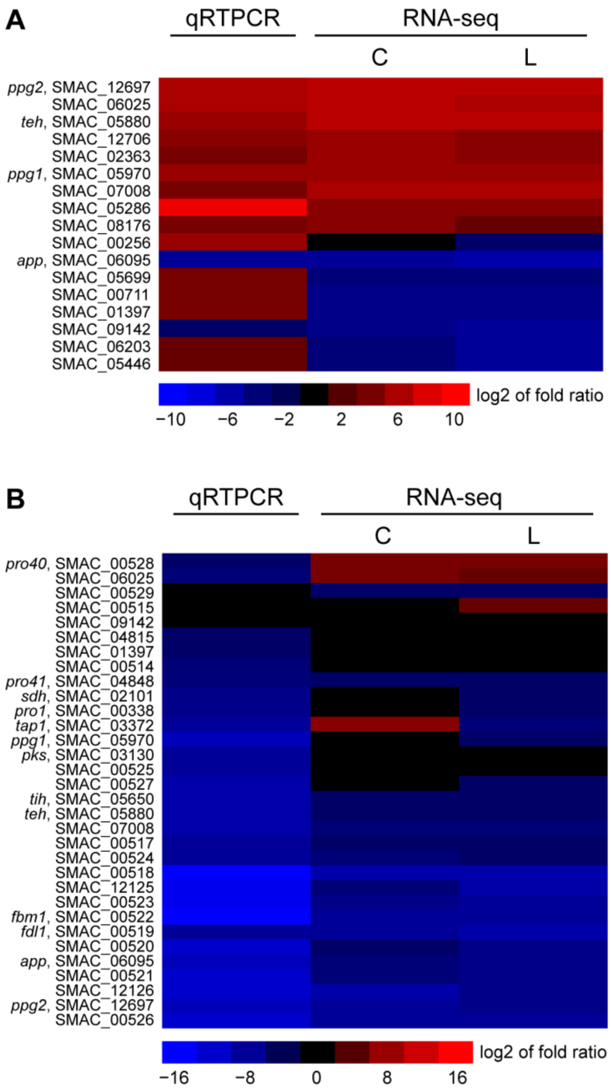

Figure S7. Transcript analysis of selected genes by quantitative real time PCR and RNA-seq. Hierarchical clustering of $\log_2$ of fold ratios for wild type protoperithecia/sexual mycelium (A) and vegetative/sexual mycelium (B). Letters C and L indicate classic (C) and LOX (L) analysis of gene expression from RNA-seq data. Quantitative real time PCR experiments were done at least twice for each gene with independent biological replicates. Quantitative real time PCR experiments in (A) were performed in this study, data in (B) were from this study for *SMAC_01397*, *SMAC_06025*, *SMAC_07008*, *SMAC_09142*, *ppg1*, *ppg2*, and *pro1*, and from previous experiments for the other genes [9, 41, 53, 73]. Note that growth conditions for total vegetative and sexual mycelia for real time PCR experiments were in defined medium, whereas for RNA-seq analysis, RNA from mycelia grown in defined medium and cornmeal medium were pooled. However, the genes investigated here are mostly involved in development or strongly differentially regulated depending on the developmental stage, and therefore not expected to be greatly dependent on the growth medium. Hierarchical clustering and heatmap generation were done in R. Gene names are given for genes that were previously shown to be involved in or differentially regulated during development.

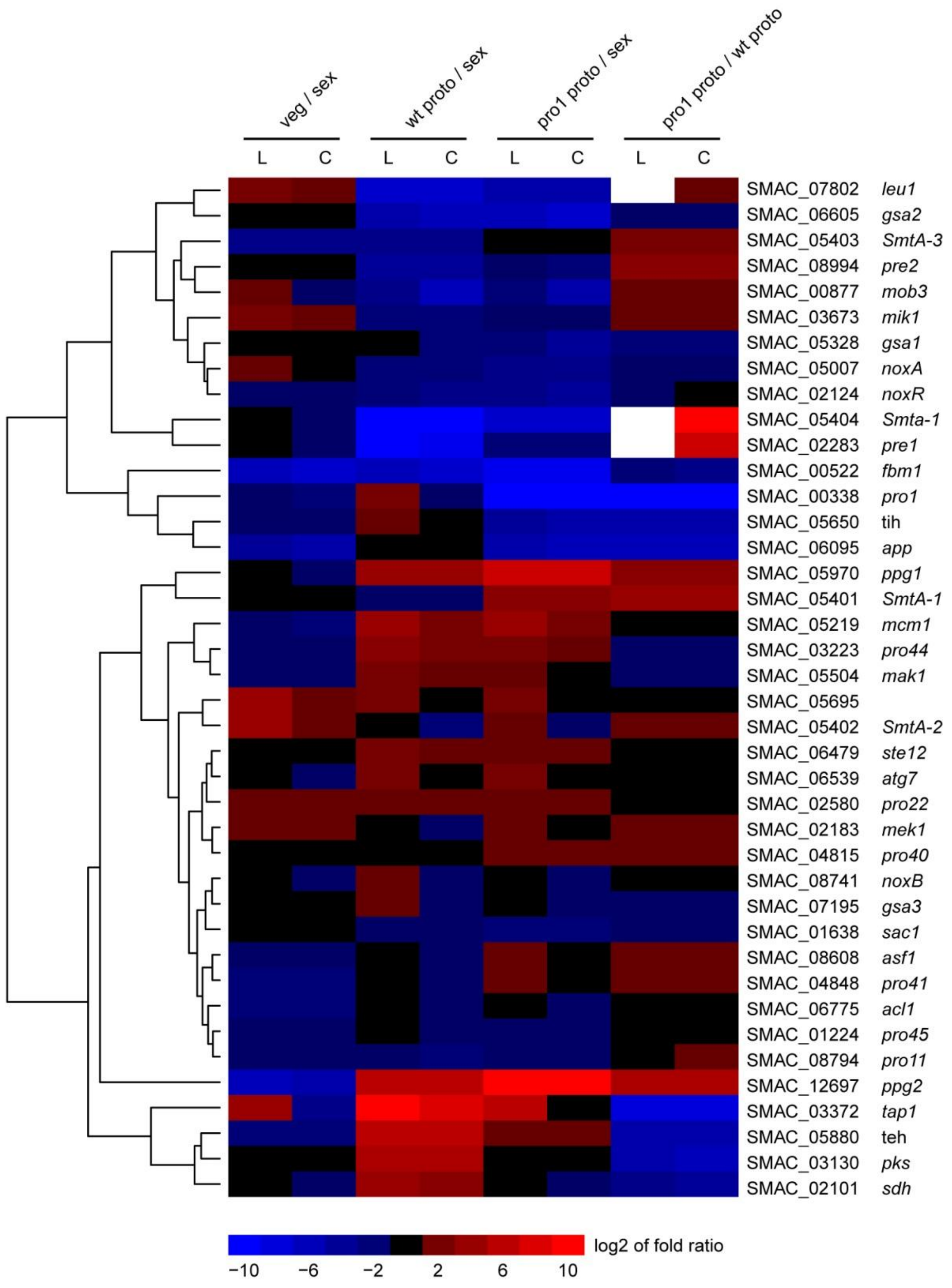

Figure S8. Expression of known developmental genes. Hierarchical clustering of $\log_2$ of fold ratios as determined by classic (C) and LOX (L) analysis. Log_2 ratios < -10 or > 10 were set to -10 and 10, respectively, to make for better scaling visibility. Hierarchical clustering and heatmap generation were done in R.

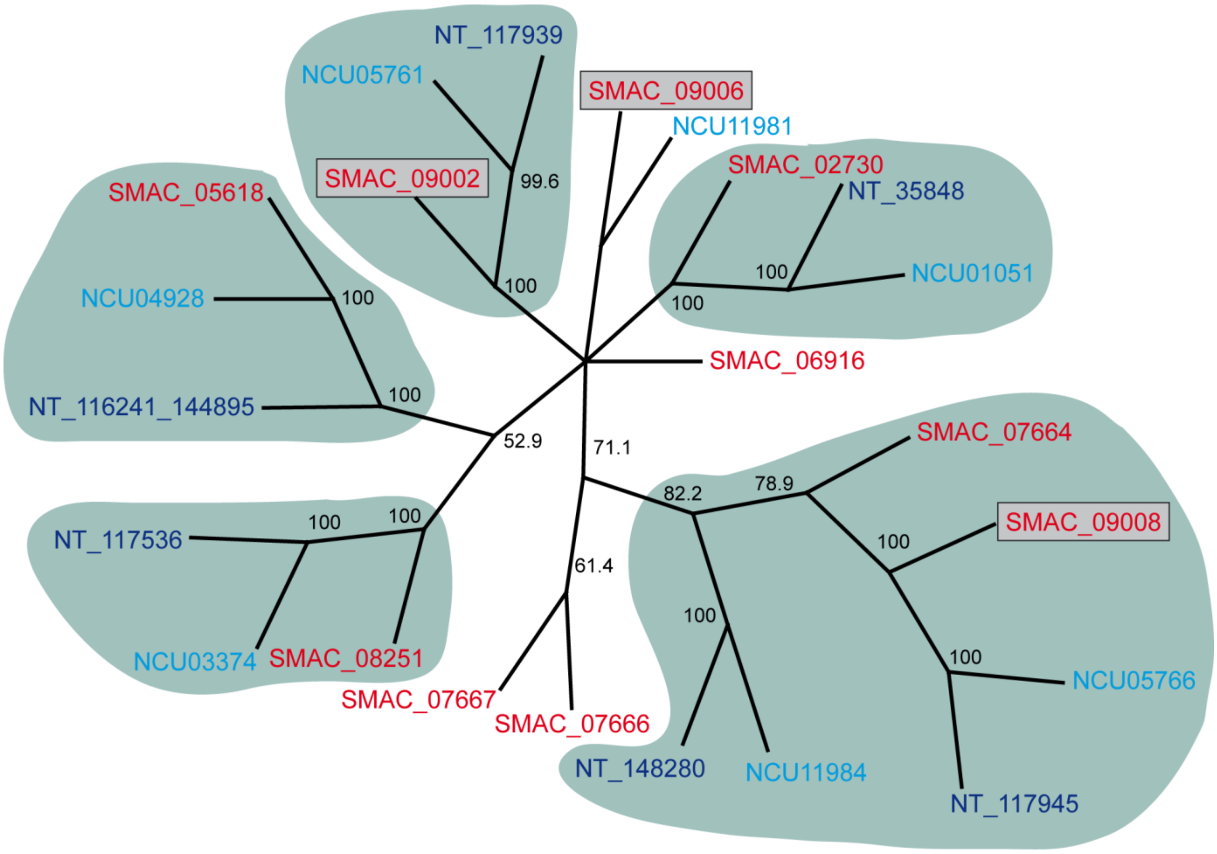

Figure S9. Phylogenetic tree of all DUF3328 proteins from *Sordaria macrospora* (red, genome version 02), *Neurospora crassa* (light blue, data from http://www.broadinstitute.org/annotation/genome/neurospora/MultiHome.html) and *Neurospora tetrasperma* (dark blue, data from http://genome.jgi-psf.org/Neute_matA2/Neute_matA2.home.html). The tree was calculated with neighbor joining with 10.000 bootstrap replications, bootstrap support in % is given at the branches. The three *S. macrospora* genes that are physically clustered within the genome are shown in grey boxes. Interestingly, the three clustered DUF3328-containing genes are not part of a closely related paralogous group, but are present on distant braches of the phylogenetic tree. Furthermore, the DUF3328-family seems to be expanded in *S. macrospora* with 10 genes in contrast to six and seven genes in *N. crassa* and *N. tetrasperma*, respectively.

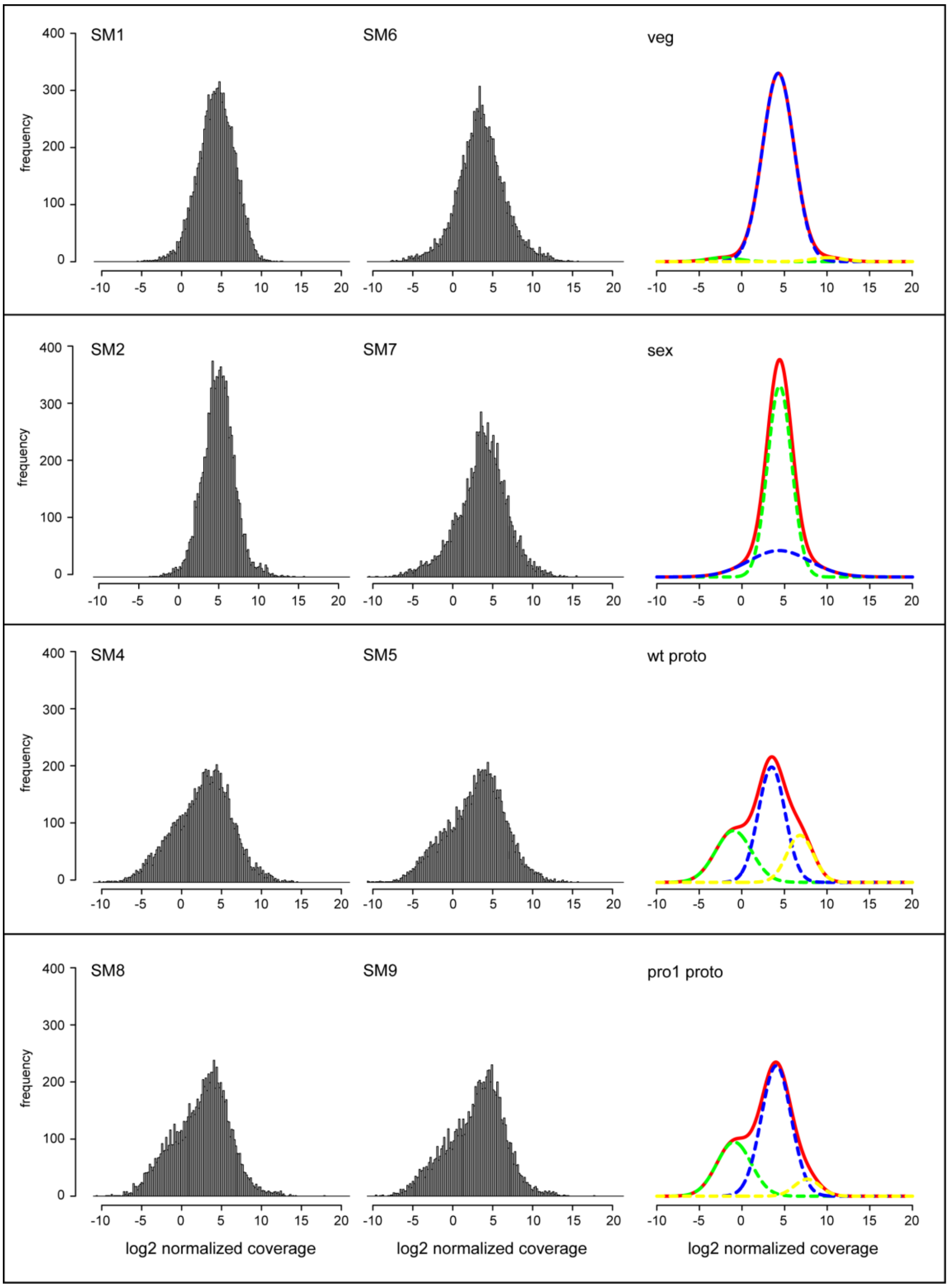

Figure S10. Distribution of gene expression levels. Histograms of log_2 of coverage (normalized to coverage per kilobase per million counted bases) for each locus tag for each sample (left, midle), and estimated frequency distribution functions for the mean for each condition (right). In case of zero coverage, log_2 coverage was set to -13 (otherwise all log_2 values > -13) and was not used here. The distribution function (red) for each condition could be dissected into components. The components (blue, green and yellow lines) are normal distributions with varying means and variances that make up different proportions of the observed distribution. Estimation of mixtures was done with the mclust package from R [66] and manual curve adjustments.

2. Supplemental Tables

Table S1. Transcription factors among the genes with top 500 read counts and their homologs in *Neurospora crassa* and *Fusarium graminearum*. Locus tag numbers are given for the *S. macrospora* (*S.m.*) genes and their homologs in *N. crassa* (*N.c.*), and *F. graminearum* (*F.g.*) with e-values for the best BlastP hit. Described phenotypes of knockout strains for the corresponding genes are from the Neurospora knockout project [55] (http://www.broadinstitute.org/annotation/genome/neurospora/MultiHome.html) for *N. crassa* and from the *Fusarium graminearum* Transcription Factor Phenotype Database [56] (http://ftfd.snu.ac.kr/FgTRPD) for *F. graminearum*, and from [54] and [9] for *S. macrospora mcm1* and *pro44*, respectively; n.d., no knockout phenotype described.

A. transcription factors among top500 genes occuring in wt proto and pro1 proto (therefore most likely not or not completely dependent on pro1)

***S.m.* locus_tag**	***S.m.* gene**	***S.m.* knockout phenotype for sexual development**	***N.c.* locus_tag**	***N.c.* gene**	**e-value *S.m.* vs. *N.c.***	***N.c.* knockout phenotype for sexual development**	***F.g.* locus_tag**	**e-value *S.m.* vs. *F.g.***	***F.g.* knockout phenotype for sexual development**
SMAC_00418		n.d.	NCU03033		$2*10^{-171}$	n.d.	FGSG_00352	$4*10^{-072}$	normal
SMAC_00439		n.d.	NCU00499	*ada-1*	0.0	few protoperithecia and perithecia, no ascospores	FGSG_00515	0.0	slightly delayed perithecial maturation
SMAC_01754		n.d.	NCU04390	*col-22*	0.0	normal	FGSG_09594	0.0	normal
SMAC_03124		n.d.	NCU03593	*kal-1*	0.0	normal	FGSG_09019	$3*10^{-180}$	reduced number of perithecia, delayed perithecial maturation
SMAC_05219	*mcm1*	no perithecia and ascospores	NCU07430		$3*10^{-114}$	n.d.	FGSG_08696	$1*10^{-083}$	reduced number of perithecia, delayed perithecial maturation, no ascospores
SMAC_05375		n.d.	NCU01924		0.0	small protoperithecia, no perithecia and ascospores	FGSG_08924	$4*10^{-041}$	normal
SMAC_06421		n.d.	NCU06095		0.0	normal	FGSG_06356	0.0	increased number of perithecia, fertile

***S.m.* locus_tag**	***S.m.* gene**	***S.m.* knockout phenotype for sexual development**	***N.c.* locus_tag**	***N.c.* gene**	**e-value *S.m.* vs. *N.c.***	***N.c.* knockout phenotype for sexual development**	***F.g.* locus_tag**	**e-value *S.m.* vs. *F.g.***	***F.g.* knockout phenotype for sexual development**
SMAC_07774		n.d.	NCU03536		$2*10^{-155}$	n.d.	FGSG_05171	$2*10^{-053}$	no perithecia and ascospores
SMAC_08084		n.d.	NCU00116		$8*10^{-159}$	n.d.	FGSG_02608	$2*10^{-097}$	delayed perithecial maturation
SMAC_08565		n.d.	NCU07952		0.0	n.d.	FGSG_01341	$6*10^{-158}$	reduced number of perithecia, maturation delayed, abnormally shaped ascospores, no ascospore discharge
SMAC_09436		n.d.	NCU11358		0.0	n.d.	FGSG_04203	$6*10^{-171}$	normal
SMAC_09459		n.d.	NCU08807		0.0	n.d.	FGSG_09715	$6*10^{-125}$	normal
SMAC_04294		n.d.	NCU02724		0.0	normal	FGSG_01307	$3*10^{-139}$	delayed perithecial maturation, abnormal shape of ascospores, reduced ascospore discharge
SMAC_02795		n.d.	NCU03064		0.0	n.d.	FGSG_01183	0.0	n.d.

B. transcription factors among top500 genes occuring in wt proto only (might therefore to some degree be dependent on pro1)

S.m. locus_tag	*S.m.* gene	*S.m.* knockout phenotype for sexual development	*N.c.* locus_tag	*N.c.* gene	e-value *S.m.* vs. *N.c.*	*N.c.* knockout phenotype for sexual development	*F.g.* locus_tag	e-value *S.m.* vs. *F.g.*	*F.g.* knockout phenotype for sexual development
SMAC_00425		n.d.	NCU03043		0.0	normal	FGSG_07052	$2*10^{-055}$	reduced number of perithecia
SMAC_02359		n.d.	NCU01629		0.0	normal	FGSG_04293	$6*10^{-062}$	normal
SMAC_03223	*pro44*	submerged protoperithecia, no perithecia and ascospores	NCU01154	*sub-1*	0.0	submerged protoperithecia, no perithecia and ascospores	FGSG_09992	$3*10^{-014}$	no perithecia and ascospores
SMAC_03952		n.d.	NCU01252		0.0	n.d.	FGSG_07456	0.0	n.d.
SMAC_06113		n.d.	NCU03938		0.0	normal	FGSG_08626	3*10-124	delayed perithecial maturation, abnormal shape of ascospores, reduced ascospore discharge
SMAC_07526		n.d.	NCU04628		0.0	normal	FGSG_13711	2*10-045	no perithecia and ascospores
SMAC_09009		n.d.	NCU05767		0.0	normal	FGSG_00774	8*10-023	normal

Table S2. Comparison of results from RNA-seq and microarray analysis. Differential gene expression between sexual and vegetative mycelium was compared for genes with expression ratios in the RNA-seq analysis and published microarray data (9). Note that mycelia for RNA extraction for microarray analysis were grown in synthetic medium, while for RNA-seq analysis, RNA from mycelia grown in synthetic medium and complete medium was combined (for both sexual and vegetative mycelium) to maximize the number of expressed genes. This might lead to a lower number of differentially expressed genes, because signals for genes that are only differentially expressed when grown in a certain medium might be quenched in this type of analysis. However, this might indicate that genes that are differentially regulated in both array and RNA-seq experiments are more likely to be affected by sexual development than by the growth medium.

	significantly diff. expression*[1]	expression tendency*[2]
Genes with expression data in both experiments:	7136	7136
Genes not differentially expressed:	6899	2922
Genes upregulated in both experiments:	1	330
Genes downregulated in both experiments:	14	405
Genes upregulated only in array exp.:	54	908
Genes downregulated only in array exp.:	116	1269
Genes upregulated only in the RNA-seq exp.:	1	932
Genes downregulated only in the RNA-seq exp.:	51	370

*[1]genes with significantly differential expression according to the thresholds that were used in the respective analyses. Thresholds for significantly differential expression were higher for the RNA-seq data than those used in previous microarray analyses.

*[2]genes with a tendency towards differential expression (ratio >1.5 or <0.67 for both types of experiment).

Table S3. Oligonucleotides used in this study.

name	sequence 5' - 3'	remarks
T7N9	GGCCAGTGAATTGTAATACGACTCACTATAGGGAGGCGGNNNNNNNNN	RNA amplification
oligo-dT(24)-anchored	tttttttttttttttttttttttt(a/c/g)(a/c/g/t)	
app-for2	GGAGATAGCTGGAGGGCTGA	quantitative real time PCR
app-rev2	ATCTCGGGCTGACTTCCATC	
ppg1-for	CTCCGTGACACCACCTTCAG	
ppg1-rev	GGAGGCATAGCGCTTCCA	
ppg2for	CGGTATCTCGCCTCTCAACGT	
ppg2rev	GTTGTGCTCCCATTGTGCAGA	
SMAC_00256-for	cgccttgcgagttgtatttg	
SMAC_00256-rev	acacgcggttcaaaagtcaa	
SMAC_00711-for	ctttggttcatcggtgatgg	
SMAC_00711-rev	tgttgcctcaaaccaaaacg	
SMAC_01397-for	ataagtgccgcccgtatgtc	
SMAC_01397-rev	ccccattatgtgccaatcct	
SMAC_02363-for	ggtaatccggcgttcttgat	
SMAC_02363-rev	tacgaagccatccgtccttt	
SMAC_05286-for	ctggttgggggacatttacg	
SMAC_05286-rev	agaacgccatttcccttgtg	
SMAC_05446-for	cgaggctttcaccgactacc	
SMAC_05446-rev	ttggcagcagacttggtgac	
SMAC_05699-for	gagggaagaggccggatact	
SMAC_05699-rev	caacctccgcctttttcttg	
SMAC_05880-for	TAAGGGCATCACGGTCAATG	
SMAC_05880-rev	TGCTCAGCCATCATCCTCTC	
SMAC_06025-for	CGATGGGGTTCAAGTGTGTG	
SMAC_06025-rev	TGACCACGTCCATCTTCAGC	
SMAC_06203-for	cgagaagggtgcttcctacg	
SMAC_06203-rev	ggcatcgaagatggaagagg	
SMAC_07008-for	tttcctccgtgacgttgaga	
SMAC_07008-rev	ctctaatggccaccgagacc	
SMAC_08176-for	attgtccggttgaggaggaa	
SMAC_08176-rev	ggaaatcaggcggaaagatg	
SMAC_09142-for	cgcaatggacgagttgttgt	
SMAC_09142-rev	gtcacgaccccttctcttgc	
SMAC_12706-for	gcgacagcaaaaggatgatg	
SMAC_12706-rev	gttgtgtcggcgatcaagag	

3. Supplemental Methods

Method S1. UTR (untranslated region) predictions from RNA-seq data.

UTRs were predicted according to the principle shown in Figure S1 with custom-made Perl scripts. The algorithms used to predict 5' and 3' UTRs were implemented as subroutines (sub) and can be described as follows:

a) sub search_for_slope

sub to search for sloping read counts (indicative of end of transcript) in given RNA-seq data. It takes an array with count data (must be sorted previously in correct order) and returns position of putative UTR (minimum) with respect to length of input array. Conditions to search for in windows of 20 nt (overlapping sliding window analysis): slope of < -1.3, and counts at beginning of slope more than 5x the counts at end of slope, and slope of next window again larger and previous window same or larger (i.e. find the steepest slope), and the number of reads at the beginning of one window has at least once reached the number of average counts for this CDS. Also, search is stopped if reads at the beginning of one window have once reached the number of average counts and afterwards drop to lower than 0.2x the average counts (without finding a UTR) or dropped to <=1 counts (even without reaching average counts previously). These conditions are specifically set for reads with a 3' bias where the peak of reads can be after the end of the CDS.

b) sub search_for_slope_full_coverage

sub to search for sloping read counts (indicative of end of transcript) in given RNA-seq data. It takes an array with count data (must be sorted previously in correct order) and returns position of putative UTR (minimum) with respect to length of input array. Conditions to search for in windows of 20 nt (overlapping sliding window analysis): slope of < -1.3, and counts at beginning of slope more than 5x the counts at end of slope, and slope of next window again larger and previous window same or larger (i.e. find the steepest slope). The difference to the subroutine search_for_slope is that here search is stopped if the number of counts at the beginning of a window is outside 0.2-5 x the average number of counts for the CDS. This is useful if the coverage of each transcript is even (no 3' or 5' bias in the reads), because then, an upward slope might indicate the beginning/end of another transcript. In data with end bias, this subroutine is not useful, because here the reads might cluster in a bell-shaped curve after the end of the CDS.

c) sub search_for_slope_gradient_to_zero

sub to search for sloping read counts (indicative of end of transcript) in given RNA-seq data. It takes an array with count data (must be sorted previously in correct order) and returns position of putative UTR (minimum) with respect to length of input array. Conditions to search for in windows of 100 nt (overlapping sliding window

analysis): slope of < -1, and counts at beginning of slope more than 3x the counts at end of slope, and the reads at the end of the slope < 10 (i.e. no more coverage beyond the transcript save background), and slope of next window again larger and previous window same or larger (i.e. find the steepest slope), and the number of reads at the beginning of one window has at least once reached the number of average counts for this CDS. Also, search is stopped if reads at the beginning of one window have once reached the number of average counts and afterwards drop to lower than 0.1x the average counts (without finding a UTR) or dropped to <=10 counts (even without reaching average counts previously). This condition is specifically set for reads with a 3' bias where the peak of reads can be after the end of the CDS. This sub is for finding gentler, longer slopes when the search for the more steep slopes has failed.

d) sub search_for_coverage

This sub is to be used if the search for slope fails (e.g. if the slope is not steep enough). This sub searches for consecutive coverage of at least 0.5x average coverage, i.e. it can find a minimum UTR. A UTR is only given as a result if the search stops before reaching the end of the upstream/downstream region which is in most cases the beginning of a new CDS or other annotated feature and transcripts at this end are most likely derived from this gene. This should prevent UTRs to be found in case of continuous coverage between two genes (where it cannot be decided where one UTR starts and the other ends). To prevent spurious reads (background) to be taken into account for UTR determination, a UTR is only calculated if the average counts are >= 50.

Method S2. Annotation of novel gene models based on RNA-seq data.

For the genome version 02, we annotated 125 novel genes based on RNA-seq patterns of spliced reads that are evidence of processed transcripts, and improved the exon-intron structure of 869 predicted genes (Figure S2). Altogether, ~1,000 genes were improved or newly annotated which corresponds to ~10 % of all genes in the *S. macrospora* genome (publicly available in genome version 2, CABT02000001-CABT02001583, http://c4-1-8.serverhosting.rub.de/public/). There could be additional not yet annotated genes that are not spliced, because for the annotation in genome version 2, new genes were derived from splice sites only (not from regions that showed some read coverage, but no evidence for spliced reads). This approach was chosen, because it seems to be a “safer” way to ensure that the annotated genes are “real” genes and not just spurious transcripts. Also, a threshold of at least 50 spliced reads to support a splice site was required, and this could probably be lowered; however, for the present annotation, a rather conservative approach was chosen to avoid calling false-positive "genes".
